# Sex Influences the Genetic Structure of Greenland Halibut in the North Atlantic

**DOI:** 10.1002/ece3.70822

**Published:** 2025-02-10

**Authors:** Daniel Estévez‐Barcia, Denis Roy, Mikko Vihtakari, Davíð Gíslason, Martin Lindegren, Asbjørn Christensen, Laura Wheeland, Margaret Treble, Julio Úbeda, Adriana Nogueira, Kevin Hedges, Áki Jarl Láruson, Alejandro Mateos Rivera, Geir Dahle, Jon‐Ivar Westgaard, Bjarki Elvarsson, Lise Helen Ofstad, Elvar H. Hallfredsson, Ole Thomas Albert, Jesper Boje, Torild Johansen

**Affiliations:** ^1^ Greenland Institute of Natural Resources Nuuk Greenland; ^2^ Department of Natural Resource Sciences McGill University Montreal Quebec Canada; ^3^ Institute of Marine Research Tromsø Department, Fram Centre Tromsø Norway; ^4^ Matis Ltd. Reykjavík Iceland; ^5^ DTU Aqua, Department of Aquatic Resources Henrik Dams Allé Lyngby Denmark; ^6^ Fisheries and Oceans Canada Northwest Atlantic Fisheries Centre St. John's Northwest Atlantic Canada; ^7^ Fisheries and Oceans Canada, Freshwater Institute Winnipeg Manitoba Canada; ^8^ Marine and Freshwater Research Institute Hafnarfjörður Iceland; ^9^ Faroe Marine Research Institute Tórshavn Faroe Islands

**Keywords:** adaptation, genetic sex determination, population genetics, *Reinhardtius hippoglossoides*, sex‐biasses

## Abstract

Greenland halibut ( *Reinhardtius hippoglossoides* ) is a commercially important species in the North Atlantic whose spatial population structure has not yet been fully determined across its entire range. We genotyped individuals from across the North Atlantic using a subset of informative single nucleotide polymorphic (SNP) markers to assess their usability as a SNP panel. We assessed whether these purportedly structured SNPs had any association with sex. We found several of these loci to be in sex‐determining chromosomes and that their inclusion generated genetic structure mainly in males. The population structure without the sex‐associated SNPs was weak and followed an isolation‐by‐distance pattern, likely with a large regional population on each side of the North Atlantic. We discuss how different sex ratios in the samples and/or an evolving sex‐determination system in this species likely caused the inclusion of sex‐associated loci in the panel. We found suggestive evidence of polymorphisms at sex‐determining chromosomes differentiating males on east and west locations, indicating evolution of the sex‐determination system. These results highlight the importance of documenting sex‐based differences in genetic studies and call for a better understanding of genomic architecture to understand sex‐determination systems across the whole distribution of sexually dimorphic species.

## Graphical abstract

Greenland halibut appears to be divided in two major units in the North Atlantic. However, previous research may have confounded sex effects and we demonstrate here the impact of including sex‐associated markers.

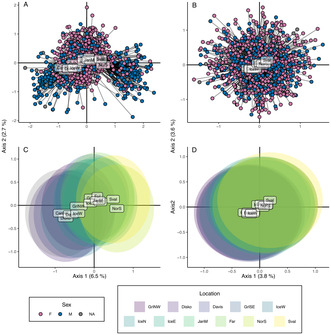

## 1 Introduction

Population genetics and genomics in fisheries management are typically focused on defining evolutionary or ecologically independent units that show some degree of reproductive isolation, which can in turn be used to define independent management regions, or stocks (Fraser and Bernatchez 2001; Ovenden et al. 2015). Thanks to the advances in sequencing technology, there has been a change in paradigm, from assuming virtually no population structure in marine species, to detecting subtle but significant differentiation (Waples 1998; Hauser and Carvalho 2008; Höglund 2009; Gagnaire et al. 2015). Some examples include population structure associated with environmental variables such as temperature (Bradbury et al. 2010), spawning times (Fuentes‐Pardo et al. 2024), and dispersal capacity (van Wyngaarden et al. 2017). These insights have cast light onto the evolutionary history of marine species, facilitating conservation and management strategies, especially considering the risks of climate change (see review in Nielsen et al. 2009) and highlighting that genomic rather than genetic studies provide higher resolution data that grants more accurate knowledge of intraspecific diversity. These studies also facilitate the development of single nucleotide polymorphism (SNP) panels that can be readily used to inform management and allow monitoring of stocks (Andersson et al. 2024). SNP panels are designed to amplify the difference between discrete units, such as spawning groups, and offer a cost‐effective alternative to whole‐genome sequencing approaches.

Our understanding of genetic diversity in several economically important species has evolved, particularly when considering non‐neutral signatures. Population structure in Atlantic herring was previously considered weak (Mariani et al. 2005), but more in‐depth studies detected a strong difference between spawning groups, which in turn were related to environmental variation (Fuentes‐Pardo et al. 2024). Similarly, horse mackerel genetic structure contrasts markedly whether using neutral or adaptive loci (Fuentes‐Pardo et al. 2023). Additionally, the interpretation of genetic structure can also be impacted by the inclusion of sex‐determining loci. Benestan et al. (2017) found that including just a handful of sex‐linked markers can markedly skew genetic structure, leading to incorrect interpretations. In fish species, where unequal sex ratios are common (e.g., Klimley 1987; Luckenbach et al. 2009; Morán, Labbé, and García de Leaniz 2016), incorporating such signals is very likely if sampling design does not account for this bias. Consequently, it is also important to understand the genetic bases of sex determination. Both Greenland halibut ( *Reinhardtius hippoglossoides* ) and Atlantic halibut ( *Hippoglossus hippoglossus* ) evolved from a common ancestor with Pacific halibut ( *H. stenolepis* ), which dispersed toward the North Atlantic after the opening of the Bering Sea. However, the genetics of sex determination differs between these species with Atlantic and Greenland halibut exhibiting an XY chromosomal sex‐determination system (Einfeldt et al. 2021; Ferchaud, Mérot et al. 2022), and Pacific halibut showing a WZ determination system (Jasonowicz et al. 2022). Hence, these species are a promising clade in which to study the evolution of sex determination. Moreover, understanding the genetic architecture of sex in these species can provide valuable tools for fisheries research, including non‐invasive methods to determine individual sex (e.g., Weise et al. 2023).

Greenland halibut is a valuable marine species within its circumpolar distribution (Vihtakari et al. 2021). It is a large predatory flatfish that exhibits demersal and pelagic behaviors, spending up to 20% of its time in pelagic habitats (Albert et al. 2011). Diet varies with size and geographical location, with smaller individuals feeding mainly on small crustaceans, shifting as they get larger toward fish, including capelin, redfish, and Atlantic cod (Hovde, Albert, and Nilssen 2002; Solmundsson 2007; Dwyer, Buren, and Koen‐Alonso 2010; Tremblay‐Gagnon et al. 2023). The occupied depth distribution also changes with size, with larger and older individuals inhabiting deeper waters (Godo and Haug 1989; Jørgensen 1997; Sohn, Ciannelli, and Duffy‐Anderson 2010; Nogueira, Paz, and González‐Troncoso 2017). Previous studies indicate multiple spawning areas in the Atlantic, including the Gulf of St. Lawrence (Domínguez‐Petit, Ouellet, and Lambert 2013), the Flemish Cap (Junquera and Zamarro 1994), the Davis Strait (Jensen 1935; Bowering and Brodie 1995), the slopes southwest of Iceland (Magnússon 1977), and the Barents Sea (Albert et al. 2001). There are several purported nursery grounds in the North Atlantic, but the evidence is strongest at two locations: the northern part of Store Hellefisk Banke and Disko Bay (Riget and Boje 1988), and the northern Barents Sea (Albert and Vollen 2015). No nursery grounds have yet been identified for the West Nordic (WN) stock (East Greenland, Iceland and Faroe Islands; Boje and Hjörleifsson 2000). Despite considerable research, resolving Greenland halibut population structure across its distribution has been difficult due to its complex life history and migratory behavior (Vihtakari et al. 2022), elusive spawning and nursery grounds (Gundersen et al. 2013; Albert and Vollen 2015; Wheeland and Morgan 2020), and low genetic differentiation (Roy et al. 2014; Westgaard et al. 2017).

Genetic structure in Greenland halibut has been investigated using various markers (allozymes‐ Fairbairn 1981; Riget, Boje, and Simonsen 1992; Igland and Nævdal 2001; Wojtasik et al. 2021; mtDNA‐ Vis et al. 1997; Orlova et al. 2019; microsatellites‐ Knutsen et al. 2007; Pomilla et al. 2008; Roy et al. 2014; Orlova et al. 2017, 2019; fewer than 100 SNPs‐Westgaard et al. 2017) and has been recently studied from a genomics perspective (Carrier et al. 2020; Gíslason et al. 2023; Ferchaud, Normandeau et al. 2022). In general, these studies describe low genetic differentiation between the Northeast and Northwest Atlantic, and while some studies support panmixia (Vis et al. 1997; Roy et al. 2014) others actually support significant structure (Knutsen et al. 2007; Westgaard et al. 2017; Gíslason et al. 2023). The genetic difference between the Atlantic and the Gulf of St. Lawrence is one of the best defined for the species (Fairbairn 1981; Carrier et al. 2020; Ferchaud, Normandeau et al. 2022) and highlights the adaptive capacity of the Greenland halibut to hypoxia (Ferchaud, Normandeau et al. 2022). There are also studies suggesting local structure within the western (Pomilla et al. 2008; Gíslason et al. 2023) and eastern regions (Wojtasik et al. 2021). The assembly of a reference genome (Ferchaud, Mérot et al. 2022) has allowed for greater capacity to detect adaptive signatures (Ferchaud, Normandeau et al. 2022), as well as the impacts of including sex‐determining markers in population structure (Carrier et al. 2020). However, these studies are limited to the comparison between the Gulf of St. Lawrence and the West Atlantic and there remains a need for studies covering wider distributions.

In this work, we used SNPs identified as informative (i.e., those showing greater genetic differentiation) from selected studies that assessed the population structure of Greenland halibut. In Gíslason et al. (2023), the authors identified several SNPs that showed genetic differentiation between nursery grounds (as proxies for spawning grounds) located in Baffin Bay, Disko Bay and Svalbard. They found significant differences between areas in the Northeast and Northest Atlantic, as well as within the West Atlantic using a large dataset of ~5600 SNPs and a smaller dataset of 90 SNPs. Building on the work by Gíslason et al. (2023), in the present study we included SNPs from the smaller set. We also included SNPs from Westgaard et al. (2017) and Berghuis et al. (in prep) which have been identified as outliers from genomic scan analyses. While the outlier SNPs identified by Berghuis et al. (in prep) were tested for association to genomic regions and environmental variables, this was not attempted in Westgaard et al. (2017). We used evidence about the sex determination in this species (Ferchaud, Mérot et al. 2022) to identify putatively sex‐associated SNPs from the raw genotype dataset. We then explored how the interpretation of population structure would vary depending on whether these SNPs were included or not. Estimates on genetic structure were calculated using a priori and a posteriori approaches, considering whether different groups were assigned to sampled locations or inferred from individual data respectively (see Miller, Cullingham, and Peery 2020). To further explore sex‐determination patterns in the Atlantic, we determined the power of SNPs located in chromosomes 10 and 21 to predict phenotypic sex. Finally, to find which factors could explain the observed genetic structure we used generalized additive models (GAM), and random forest models (RF). GAMs constitute a regression method suitable for examining non‐linear dependencies in ecological data (Wood 2017), while RF is a machine learning tool suitable for ecological applications where variables have varying contributions on response variables (Breiman 2001). We used these models to evaluate the influence of fish length and/or sex and longitude on genetic structure.

Our primary aim is twofold: (1) determining whether sex‐biases could be impacting estimates of genetic structure; and (2) how well do SNPs discovered in sex‐determining regions of the genome predict sex.

## 2 Materials and Methods

### 2.1 Sampling

**FIGURE 1:**
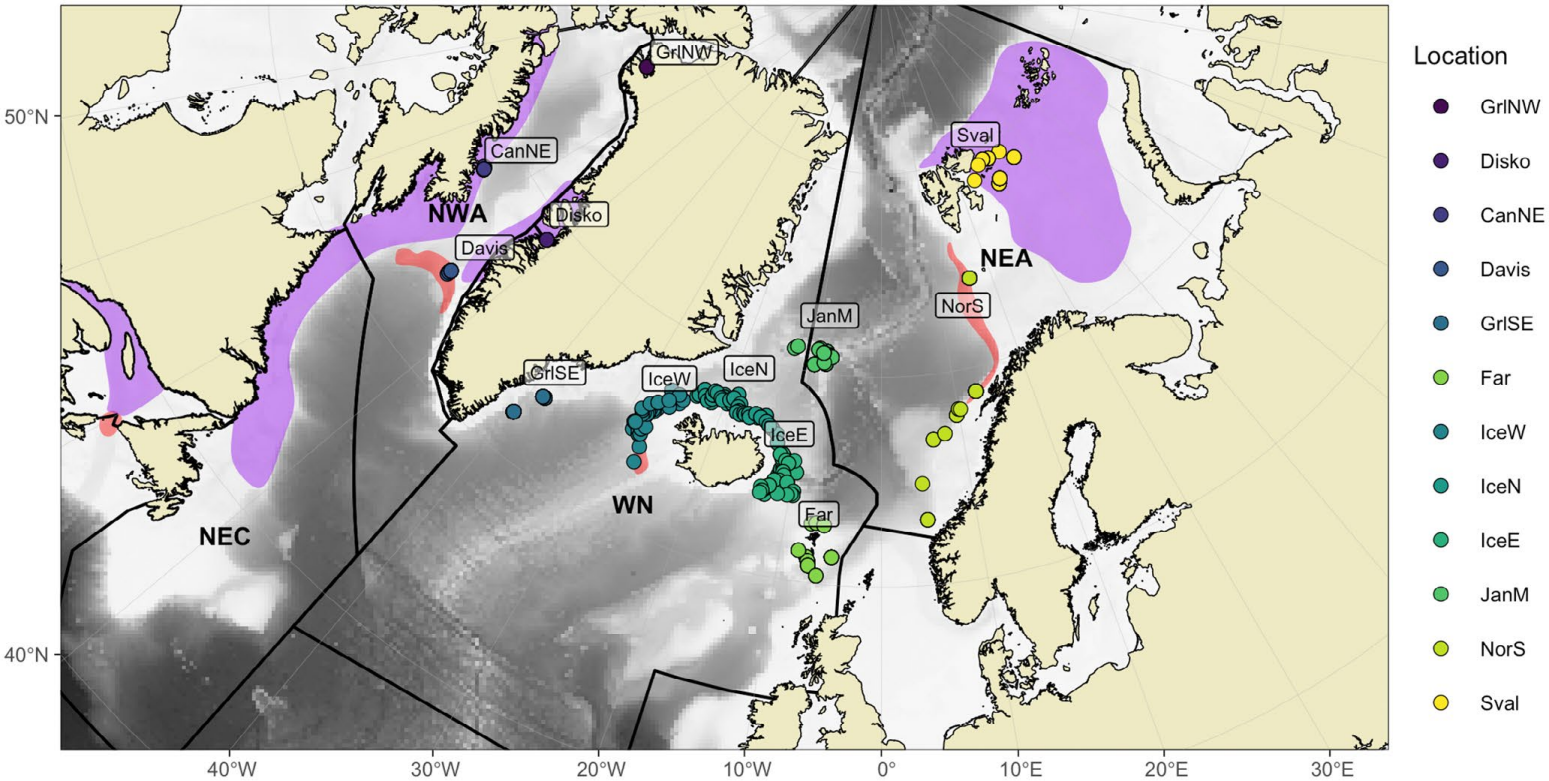
Sampling locations for Greenland halibut in the North Atlantic (acronyms are described in Table 1). Depending on coordinate availability, points may indicate the sampling of a single individual or the average for several hauls. The main nursery and spawning grounds are shown in purple and red polygons respectively. The division of the four main offshore stocks is also shown (NEC, Northeast Canada; NWA, Northwest Atlantic; WN, West Nordic; NEA, Northeast Arctic).

Sampling was conducted at 12 different locations: Northwest Greenland (Qaanaaq fjord, *GrlNW*), Disko Bay (*Disko*), Northeast Canada (Qikiqtarjuaq, *CanNE*), Davis Strait (*Davis*), Southeast Greenland (*GrlSE*), west, north, and east Iceland (*IceW*, *IceN* and *IceE*), Faroe Islands (*Far*), Jan Mayen (*JanM*), the Norwegian continental slope (*NorS*), and Svalbard (*Sval*; Figure 1, Table 1). Most samples were collected from late winter 2019 to summer 2020, but samples from Iceland and Jan Mayen were also collected in 2018. Both *Disko* and *Sval* samples were also used in Gíslason et al. (2023). In this study we have grouped the different samples into the following areas: Northwest Atlantic, NWA (*GrlNW*, *Disko*, *CanNE*, and *Davis*), West Nordic, WN, (*GrlSE*, *IceW*, *IceN*, *IceE*, and *Far*) and Northeast Arctic, NEA (*JanM*, *NorS* and *Sval*). Individuals were primarily obtained from scientific surveys conducted by the Greenland Institute of Natural Resources (GINR), the Institute of Marine Research of Norway (IMR), the Marine and Freshwater Research Institute of Iceland (MFRI), the Faroe Marine Research Institute (FAMRI), and Fisheries and Oceans Canada (DFO). Additional samples were provided by the commercial fishery under agreements with these institutions. We also provide, when possible, the depth at which individuals were collected per location (Table 1). Total length was measured to the nearest centimeter for all individuals. Sex determination was performed by visual inspection of the gonads for most individuals, except for those collected from *JanM* in 2018 and from *CanNE*, where no phenotypic sex was recorded. Tissue for genetic analysis was sampled from gill filaments or fin clips and preserved in 96% ethanol until DNA extraction.

**TABLE 1:** Greenland halibut sampling locations, their abbreviations, sampling year and season, number of individuals collected (count), mean length (cm), length range (cm), number of individuals per sex (females and males), and mean sampling depth (m). Number of individuals are reported before and after filtering.

Sample	Code	Year(s)	Month(s)	Count	Length	Length range	Females	Males	Depth
Pacific	Pac	1991–2012	Aug‐Sep	22/19	57.7	34–83	—	—	104
Qaanaaq	GrlNW	2020	Oct	100/72	63.8	56–70	72/54	27/17	350
Disko Bay	Disko	2019	Dec	95/71	25.6	18–38	51/35	43/36	358
Qikiqtarjuaq	CanNE	2019	Sep	17/13	24.5	20–30	—	—	678
Davis Strait	Davis	2020	Feb‐Jan	84/78	59.2	38–93	49/44	35/34	1039
Greenland East offshore	GrlSE	2019	Jun	90/87	65.4	52–87	73/70	17/17	—
Faroe Islands	Far	2019	Sep	94/93	58.9	37–82	82/81	12/12	505
Iceland West	IceW	2018–2019	—	78/77	67.4	39–101	44/43	32/32	841
Iceland North	IceN	2018–2019	—	147/146	56.7	35–76	117/116	29/29	608
Iceland East	IceE	2018–2019	—	73/71	58.7	41–99	57/55	15/15	535
Jan Mayen	JanM	2018–2020	Jul‐Oct	123/87	65.4	43–89	62/48	28/17	NA
Norwegian Slope	NorS	2019–2020	Apr‐Sep	211/203	52.2	36–92	111/107	100/96	707
Svalbard	Sval	2019	Sep	92/92	20.6	11–43	33/33	58/58	313

A sample (*n* = 22) from the Pacific Ocean (*Pac*; mean coordinates: 58°16′23.13″ N, 170°34′1.53″ W, Bering Sea), was obtained from a museum collection at the University of Washington, provided by the United States' National Oceanic and Atmospheric Administration (NOAA). This sample was used as an outgroup in our analysis and to assess the discriminatory power of our genetic markers, because Pacific populations are significantly different from Atlantic ones, and the value of differentiation is greater than within ocean basins (Fairbairn 1981; Orlova et al. 2017). Sex was not assessed in this sample.

### 2.2 DNA Extractions and Genotyping

DNA extraction was carried out using the E‐Z 96 Tissue DNA Kit (Omega Bio‐Teck Inc.) following the manufacturer's protocol. Individuals (*n* = 1226) were genotyped for a total of 93 SNPs selected from previous studies (17 with prefix “GrHa” from Westgaard et al. 2017; Berghuis et al. in prep; and 76 with prefix “locus” from Gíslason et al. 2023). These SNPs were selected based on outlier detection tests such as those implemented in BayeScan, PCAdapt, and LOSITAN (Antao et al. 2008; Foll and Gaggiotti 2008; Luu, Bazin, and Blum 2017) or establishing a *F_ST_* threshold (Gíslason et al. 2023). Given the geographic span of Gíslason et al. (2023), the SNPs from this study may augment the difference between Disko and Sval (both samples also included in this study), although differences within the west Atlantic were also observed with these loci. The study area in Berghuis et al. (in prep) is primarily restricted to the northwest Atlantic while in Westgaard et al. (2017) they studied samples from across the North Atlantic. The SNPs were organized into five assays, and the samples were genotyped using matrix‐assisted laser desorption/ionization time‐of‐flight mass spectroscopy (MALDI‐TOF MS; Agena Bioscience Inc., Hamburg Germany). Typer Analyzer (MassARRAY Typer, Agena BIoscience) software was used for automated genotype calling and all genotype calls were quality controlled by two independent observations.

### 2.3 Data Cleaning and Basic Genetic Stats

Loci missing more than 20% of genotypes were removed using the missingno function from the R package poppr v 2.9.3 (Kamvar, Tabima, and Grünwald 2014). Thereafter, the dataset was filtered for minor allele frequency with a threshold of 1%. Finally, individuals missing more than 20% of genotypes were removed. Hardy–Weinberg equilibrium (HWE) and linkage disequilibrium (LD) were tested using the R version of genepop v 1.1.7 (Rousset 2008). For HWE tests, Markov chain Monte Carlo (MCMC) parameters of 10,000 dememorization steps, using 20 batches and 5000 iterations per batch were used to estimate exact *p*‐values. LD was tested for each pair of loci within each location using the default statistic (likelihood‐ratio test). Exact p‐values were estimated through MCMC with 100 batches. Following Benjamini and Hochberg (1995), the false discovery rate (FDR) for multiple comparisons was evaluated in base R (R Core Team 2022), using the function *p.adjust*, in both HWE and LD. Loci consistently out of HWE and/or with significant LD across locations were removed from the analyses (one from each pair in case of LD). The level of heterozygosity and inbreeding was estimated for all loci using the function *basic. stats* from the R package *hierfstat* v. 0.5–10 (Goudet and Jombart 2022). Additionally, loci were noted for excess levels of observed heterozygosity (H_O_) using a threshold of 0.5, and for inbreeding coefficient (*F*_IS_) values greater or lower than +/−0.40 within samples. We considered the number of violations across locations as criteria for locus removal as some of these may result from low sample size or may occur only in one or two samples.

### 2.4 Identifying Sex‐Associated Loci

To identify the SNPs that could be associated with sex, we created fasta files of 1500 bp regions around SNPs with the prefix “locus,” and 150 bp around the ones with the prefix “GrHa” (sequences of 3000 and 300 bp, respectively). We then aligned these fasta files against the Greenland halibut reference genome (Ferchaud, Mérot et al. 2022) using the Basic Local Alignment Search Tool (BLASTn) available from the National Centre for Biotechnology Information (NCBI; Camacho et al. 2009). We used the whole‐genome‐shotgun contigs as the selected database. BLASTn results were recorded when coverage exceeded 90%. *E*‐values were always reported as 0, so no alignment was attributable to chance alone (Table S3). Those SNPs located in chromosomes 10 and 21 were identified as putatively sex associated, given the divergence between sexes reported in the literature for these chromosomes (Ferchaud, Mérot et al. 2022). We follow precedence by Ferchaud, Normandeau et al. (2022), whereby all regions within these two chromosomes were assumed to be sex associated. Two datasets, one with and one without putative sex‐associated SNPs, were used to estimate their influence on population structure. An additional dataset with only the SNPs located on chromosomes 10 and 21 was used to evaluate their accuracy in determining sex, relative to visually assigned sexes.

### 2.5 Effect of Sex Loci in Estimates of Population Structure

Before analyzing the data an estimate of genetic differentiation for samples collected in the same locations but over different years was estimated (*F_ST_*; Weir and Cockerham 1984). As no significant *F_ST_* values were observed in these comparisons (Table S4), samples from different years were pooled (pooled samples were used in all subsequent analyses).

We then evaluated the impact of including SNPs located in chromosomes 10 and 21 (hereafter called sex‐associated) into estimates of genetic differentiation and structure using two different genotype datasets: with and without sex‐associated loci. We compared these two datasets at a location and at an individual level. At the location level, or a priori (since different groups are predefined by sampled locations), we calculated pairwise *F_ST_* values with the *pairwise. WCfst* function in the *hierfstat* R package. To evaluate significance, we used confidence intervals (CIs) calculated by bootstrapping with 1000 permutations, as implemented in the function *boot.ppfst* from the same package. *F_ST_* estimates were considered significant if their CI did not include zero. These estimates were also plotted using Classic Torgerson's metric multidimensional scaling (i.e., principal component analysis, Torgerson 1958) enabling inference of *F_ST_* magnitudes among locations.

At the individual level (or a posteriori), we performed a principal coordinate analysis (PCoA). PCoA was performed with the *dudi.pco* function from the R package *ade4* v 1.7‐19 (Dray and Dufour 2007), on a Euclidean distance matrix calculated from individual allelic frequencies. Individual coordinates were plotted along with sample average coordinates to visually assess the geographic extent of individuals within samples. A PCoA was also undertaken using only the sex‐associated SNPs. Finally, using the dataset excluding sex‐associated loci, we estimated the number of different genetic clusters using the Bayesian clustering method implemented in the program STRUCTURE v 2.3.4 (Pritchard, Stephens, and Donnelly 2000). STRUCTURE was run with the admixture model, using correlated allele frequencies, and consisted of 300,000 burn‐in steps followed by 100,000 MCMC iterations. The range of possible genetic clusters tested (*K*), spanned from 2 to 6, with 10 replicates per *K*. To identify the most likely value of *K*, the Evanno correction was used in Structure Harvester v 0.6.94 (Evanno, Regnaut, and Goudet 2005; Earl and vonHoldt 2012). Subsequently, the files from the best supported K were combined using CLUMPP v 1.1.2 (Jakobsson and Rosenberg 2007) to account for variation among replicates.

### 2.6 Factors Explaining Observed Genetic Structure Excluding Sex‐Associated SNPs

To assess the relative importance of longitude, length, and sex in explaining differences in the probabilities of individuals being assigned to cluster 1 or 2 (STRUCTURE results), we applied generalized additive (GAM) and RF models. The same set of candidate explanatory variables were included in both methods testing the relationship:

$$P{\left(Q_{1}\right)}_{i}=a+s\left({\text{length}}_{i},{\mathrm{sex}}_{i}\right)+s\left({\mathrm{lon}}_{i},{\mathrm{sex}}_{i}\right)+{\text{siteID}}_{i}+e_{i}$$

where the response variable *P*(*Q1*) is the assigned probability of each individual *i* belonging to cluster *Q1* as a function of its length (cm) and longitude. To account for potential differences in responses between males and females we allowed the fitted relationships with length and longitude to vary with sex. In addition, to assess variations among individuals originating from sampling sites, *siteID* was introduced as a random factor. The constant *a* is the overall intercept, *s* the thin plate smoothing function for each term, and *e* is the error. Since the response variable constitutes a ratio bounded between zero and one, we used a Beta distribution during model fitting. Furthermore, the degrees of freedom of the spline smoother function (*s*) were further constrained to three knots (*k* = 3) to allow for potential non‐linearities but restricted flexibility during model fitting. To identify the best possible set of predictors we applied backwards model selection comparing full and reduced models based on AIC and likelihood‐ratio tests. In terms of RF no such model selection was done, but instead, we estimated the relative importance of each predictor. Finally, the resulting statistical relationships and derived response curves between the set of covariates and *P*(*Q1*) were compared across methods to assess the sensitivity and robustness of the model to the choice of method. The statistical modeling was conducted using the R software, version 4.2.3 using the following packages: *mgcv* v. 1.9‐1 (Wood 2011), *randomForest* v. 4.7‐1.1 (Liaw and Wiener 2002), and *MixRF* v. 1.0 (Wang and Chen 2016).

### 2.7 Association Between Phenotypic Sex and Sex‐Associated Loci

The sex‐associated loci were evaluated for their power to genetically identify the sex of individuals, in those cases where sex was visually evaluated. For this, we ran a classification RF analysis using the *randomForest* package. We evaluated an initial model with 1000 decision trees and five input variables per tree. This first model included length and sampling location, which were both identified as important in classifying the sex of individuals. We therefore removed the length variable, which is highly related to sex in this species: female Greenland halibut grow older and larger than males, and in our sample set all individuals > 75 cm were females. Also, the removal of the length variable ensured that the classification power depended solely on the SNP genotypes. To account for the effect of sampling location, we also ran different models with datasets containing samples west and east of Cape Farewell and datasets that only contained samples from NWA, WN, or NEA areas. To ensure that sex ratios did not affect the prediction model, all RF models were run using equal sample sizes for males and females using the *sampsize* option in the *randomForest* function. Observed heterozygosity for males and females per location sampled and *F_ST_* values for sex‐associated loci within location were calculated using the *hierfstat* package.

## 3 Results

### 3.1 Filtering and Basic Stats

Of the 93 loci genotyped in 1226 individuals, ten were excluded due to either being monomorphic in our sample (locus71 and locus77) or having ≥ 20% missing data (GrHa07, GrHa18, locus03, locus04, locus10, locus44, locus65, and locus72). Additionally, 117 individuals with ≥ 20% missing genotypes across all remaining loci were removed from the analyses (these were mostly within the *GrlNW* and *JanM* samples, with 28 and 37, respectively). Several loci in the *Pac* and *CanNE* samples were monomorphic (18 and 6, respectively) which is most likely due to low sample sizes from these locations (see Table 1). Three loci had a deficit of heterozygotes in almost all locations, while all other loci were in HWE (Figure S1). Linkage disequilibrium in all samples was only observed between locus42 and locus43 (Figure S2), leading to the exclusion of locus42 from further analysis. Significant linkage between loci varied among samples and remained below 2%, with no consistent pair of loci across locations.

Heterozygosity estimates across loci in all locations hovered around 0.3. Two loci had several occurrences with an observed heterozygosity > 0.5 (Figure S3, Table S1; Table S2). The overall inbreeding coefficient was −0.01 with a maximum of 0.21 for locus12, locus90, and a minimum of −0.2 for GrHa04, locus76, and locus79. Locus12 exhibited an inbreeding coefficient exceeding 0.40 in five locations (Figure S3). Most other cases where loci exceeded *F*_IS_ = 0.40 were observed in the *Pac* and *CanNE* samples, likely stemming from their smaller sample sizes (Table 1). After filtering, the dataset contained 1109 individuals genotyped at 76 SNPs.

### 3.2 Identifying Sex‐Associated Loci

BLASTn results indicated that the SNPs used in this study were distributed across most of the 24 chromosomes of the Greenland halibut genome (Ferchaud, Mérot et al. 2022; Table S3). SNPs located in chromosomes 10 and 21 represented approximately 37% of all loci included here after general filters (24 SNPs). After this, we considered two main datasets, one including (76 SNPs) and one excluding (52 SNPs) sex‐associated SNPs.

### 3.3 Effect of Sex Loci in Estimates of Population Structure

**FIGURE 2:**
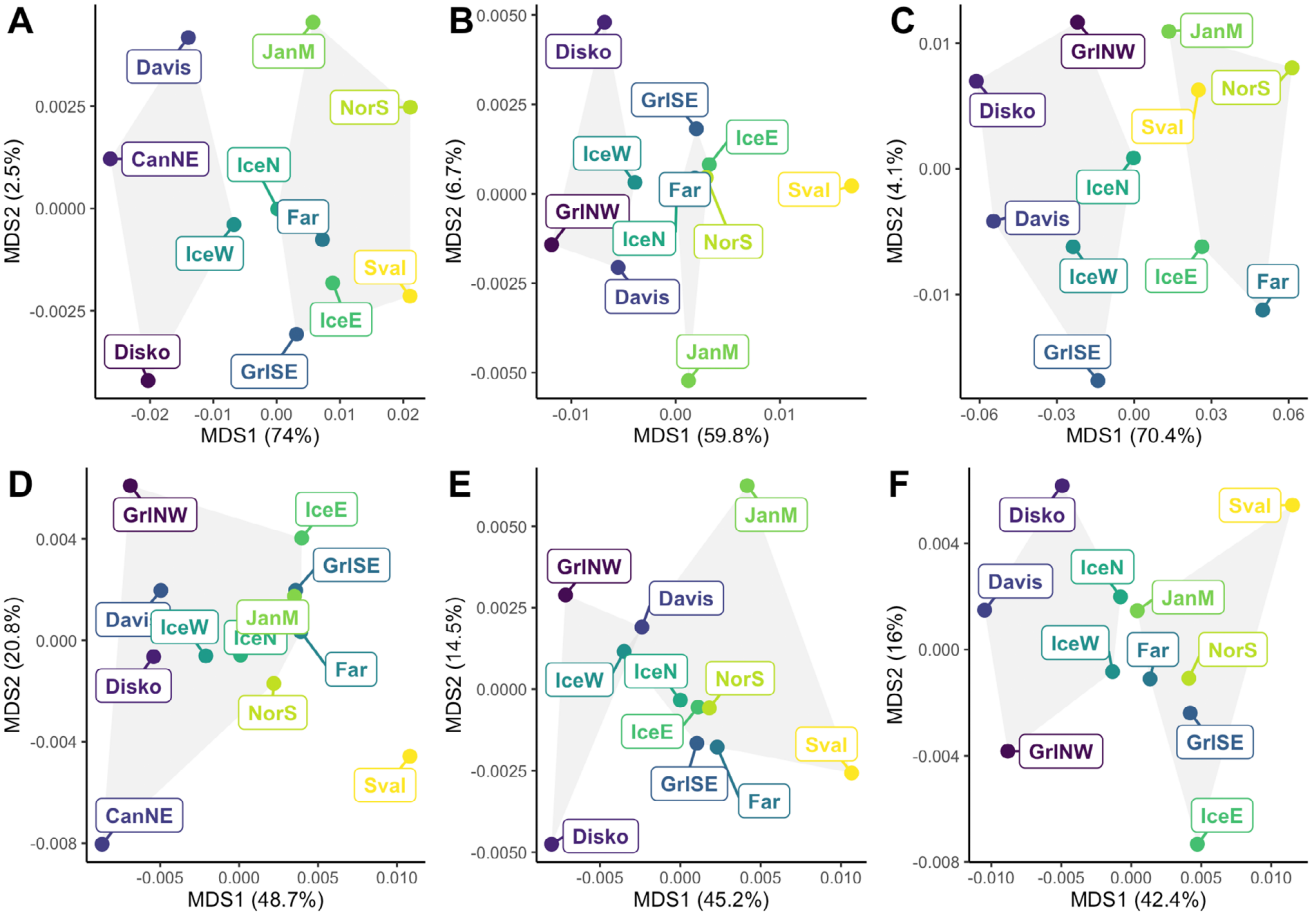
Classical multidimensional scaling (MDS) of pairwise Weir and Cockerham *F_ST_* values (points) for all samples (A and D), females (B and E) and males (C and F) with (A–C) and without (D–F) sex‐associated loci. The color of labels indicates the longitudinal gradient with westernmost locations colored blue and easternmost yellow. Gray polygons separate grouped locations acquired using hierarchical clustering. The distance between locations on the coordinate system gives an approximate *F_ST_* value. The percentage of variance explained by each dimension is indicated within brackets.

The inclusion of sex‐associated SNPs clearly altered the pattern of population structure. Multidimensional plotting of *F_ST_* based matrices showed sampled locations to group in a similar manner for all cases, with two groups including western and eastern samples respectively (Figure 2). Nonetheless, the magnitude of pairwise *F_ST_* values was noticeably higher when including sex‐associated SNPs, with dimensions in the MDS biplot explaining more variance overall. This difference in magnitude was more obvious in males than in females (Table S5, Figure 2A–C). The *Sval* sample showed more distance compared to the other locations when including sex‐associated SNPs in females (Figure 2B) and without separating by sex and excluding sex‐associated SNPs (Figure 2D). After excluding sex‐associated loci, the remaining SNPs showed the greatest differentiation between the Pacific sample and the remaining locations with a value of *F_ST_* = 0.092 ± 0.01 (SD) within a 95% CI spanning from 0.056 to 0.13. Compared with the overall North Atlantic estimate (*F_ST_* = 0.008), this was the highest estimate of genetic differentiation (Table S6).

**FIGURE 3:**
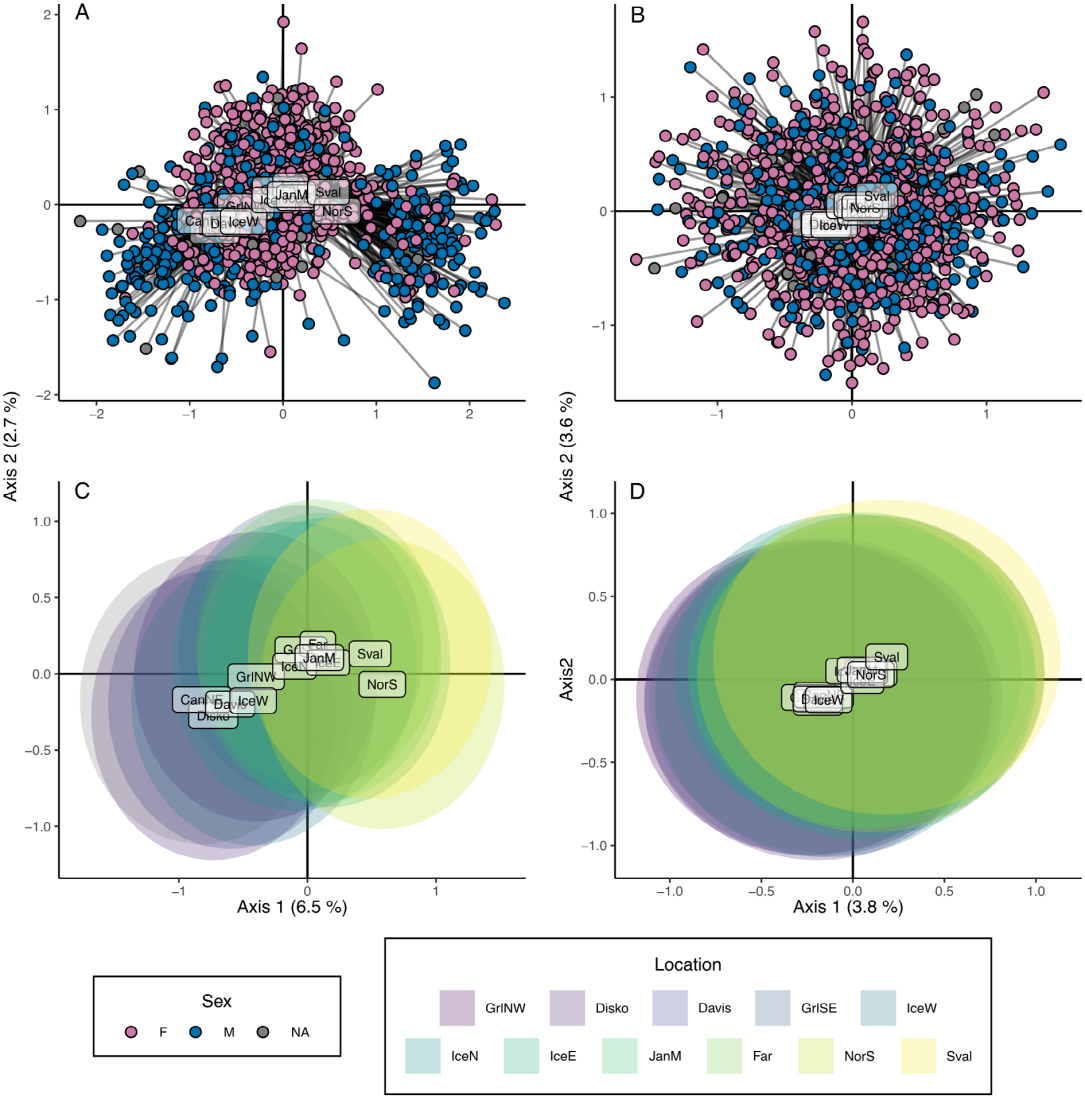
Principal coordinate analysis plots based on Euclidian distances using the datasets including (A and C) and excluding (B and D) sex‐associated loci. In all plots location labels are the coordinate's mean calculated from individuals within locations. Plots A and B show how individuals of both sexes (F, female; M, male; NA, unassigned) scatter in the coordinate space for the two first axis. Plots C and D show the Euclidian ellipses centered on each location coordinate mean.

PCoA results further confirmed the effect of including sex‐associate loci. A pattern of West vs. East population structure was more accentuated when including these SNPs than when they were excluded (Figure 3C,D). Including these SNPs led to a greater split between west and east in males (Figure 3A), showing a difference in grouping between the two sexes. In contrast, removing these loci caused individuals of both sexes to scatter homogeneously across the two‐dimensional PCoA space (Figure 3B). Note that a PCoA using only the sex‐associated markers (*n* = 24) shows the same pattern as in Figure 3A (Figure S4).

**FIGURE 4:**
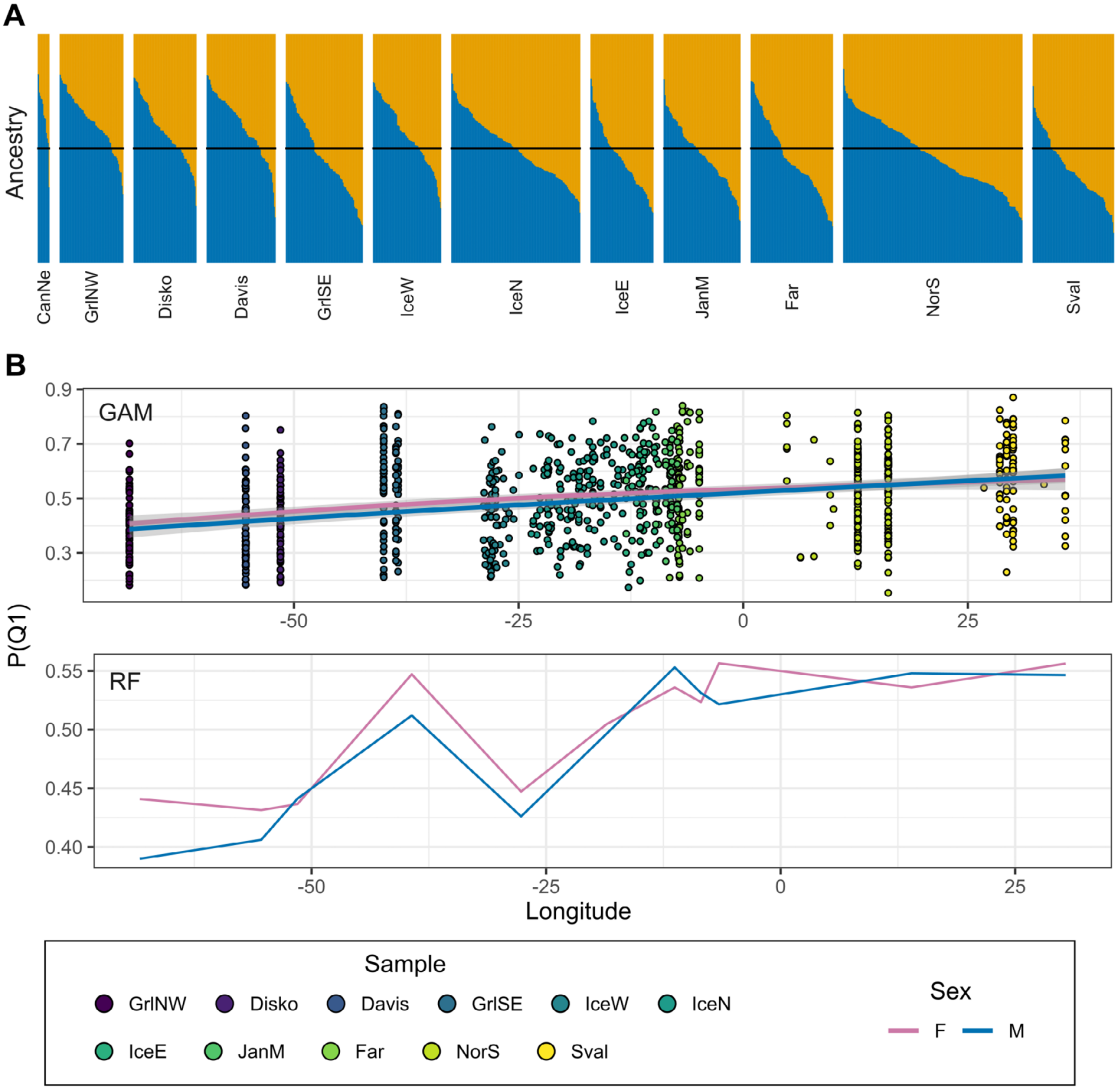
STRUCTURE plot showing the proportion of inferred ancestry per individual for *K* = 2 (A), and response plots based on Generalized Additive Model (GAM; B top) and Random Forest (RF; B bottom) showing the effect of longitude on the probability of assignment to cluster 1 for both males (blue) and females (purple). The GAM response plot shows the fitted relationships with solid lines and shaded areas representing the mean and 95% confidence intervals, respectively, and the points the observed values for each individual. In the RF response plot, the predicted probabilities across longitudes are plotted, while maintaining additional predictors at their median values. This figure does not include sex‐associated SNPs.

Evanno correction applied to all STRUCTURE runs as applied in StructureHarvester identified two clusters as the most likely number of genetic groups across the North Atlantic (Figure S5). It is important to note that *K* = 1 cannot be tested by this method, and we present the highest likelihood with *K* > 1, where we can investigate admixture patterns. Most of the samples had a high level of admixture between the two clusters and there was no clear cut between east and west samples, although the assignment level to *K* = 2 was higher in the West than in the East (Figure 4A). This result was produced after excluding sex‐associated SNPs and indicate, together with the previous described results, weak population structure when excluding sex‐associated SNPs.

### 3.4 Factors Explaining Observed Genetic Structure After Excluding Sex‐Associated SNPs

GAM and RF models explained 15.1% and 12.3% of the variance in assignment probability to one of the two clusters, respectively. Among the candidate predictors, longitude was found to be significant with a large effect size, while length was not significant and therefore removed during model selection. The final GAM (excluding length) exhibited the lowest AIC making it more likely than the models including length (Table 2). In terms of RF, longitude was also deemed the most important predictor, with a variable importance score (2.27) higher than length (1.63) and sex (0.13). Response curves between *P*(*Q1*) and longitude show consistent positive linear trends for both sexes using either GAM or RF (Figure 4B).

**TABLE 2:** Summary statistics of the final Generalized Additive Model, including the intercept and smooth terms for the retained predictors. Edf is the estimated degrees of freedom for the model smooth terms (s) (i.e., edf > 1 indicates a non‐linear relationship), and the associated chi‐squared statistics.

Component	Term	Estimate	STD error	*t*	*p*
A. parametric coefficients	(Intercept)	−0.011	0.042	−0.267	0.790

Component	Term	edf	Ref. df	*F*	*p*
B. smooth terms	s(lon, Sex)	1.906	7	781.004	0.00***
	s(pop)	7.398	10	42.312	0.00***

*Note:* Adjusted *R* ^2^: 0.139, deviance explained 0.151. −REML: −579.957, Scale est.: 1.000, *N*: 1049.

*** *p* ≤ 0.001.

### 3.5 Association Between Phenotypic Sex and Sex‐Associated Loci

**FIGURE 5:**
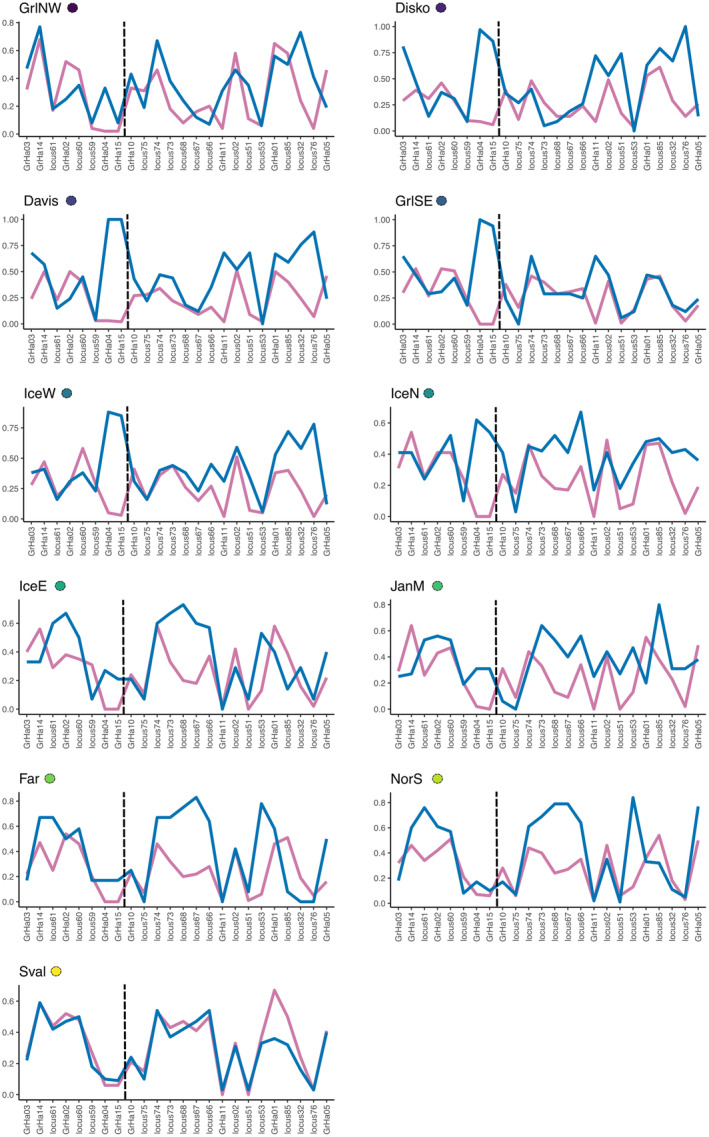
Observed heterozygosity comparisons between males (blue) and females (pink) for each sex‐associated locus within each sampled location. Notice that locations are displayed following a NW to NE geographical cline (circles next to location acronym indicate the same color scheme using throughout the paper). Dashed vertical lines separate SNPs in chromosome 10 (left) from those in chromosome 21 (right). All SNPs are organized according to their position in the chromosomes.

The SNPs located in chromosomes 10 and 21 were good predictors of sex in almost all RF models (Table 3). The classification error was in general higher in males than in females, and out of all models, the one including NEA locations reported the highest classification errors. This result is likely driven by the *Sval* sample, where these SNPs accounted for lower genetic differentiation compared to the remaining locations (Figure S6). Heterozygosity comparisons between males and females were not homogeneous across locations. For instance, heterozygosity differences between sexes were greater for loci GrHa04 and GrHa15 (males more heterozygous) in western locations (e.g., *Disko*, *Davis*, and *IceW*) than in eastern locations (e.g., *NorS*, *IceE*, *JanM*). But in eastern locations, these loci did not show such difference. Instead, other loci, like locus76 and locus53 showed greater differences in these locations, but not in the west (Figure 5).

**TABLE 3:** Sex classification error for the different random forest models predicting sex from loci on Chromosomes 10 and 21. The different models include different sample locations: Atlantic‐ all locations; west‐ all locations west of Cape Farewell, notice that this model also corresponds to the Northwest Atlantic area (NWA); east‐ all locations east of Cape Farewell; WN‐ all locations within the West Nordic area; NEA‐ all locations within the Northeast Arctic area. Number of individuals visually assigned to each sex (*N*). Classification errors correspond to the percentage of individuals whose sex prediction (from the models) did not match visual assignment.

Model	Females		Males	
	*N*	Class. error	*N*	Class. error
Atlantic	686	8.9	363	9.4
West	133	5.3	87	13.8
East	553	7.4	276	14.8
WN	365	2.7	105	4.8
NEA	188	18.6	171	23.4

## 4 Discussion

### 4.1 Accommodating Limitations

Before starting to discuss the results, it is noteworthy to acknowledge the limitations of the data and how we have accommodated for them. We note here that we used a small set of SNPs designed to separate Northwest and Northeast Atlantic and there are limitations to the strength of our conclusions regarding the population structure. The original design to identify these SNPs was based on juvenile individuals that could serve as proxies for separated genetic clusters from Disko Bay and Svalbard (the same samples were also used here), and two Canadian locations, and thus could cause ascertainment bias when considering the entire distribution. However, the lack of juveniles around Iceland (Boje and Hjörleifsson 2000), constrained the availability of reference individuals for identifying a distinct unit or units which could represent the WN stock, when designing the SNP subset (Gíslason et al. 2023). Note, however, that the lack of juveniles also functions as evidence for the lack of major spawning sites directly producing juveniles for the stock. Consequently, the resulting SNPs used here, though providing structure results on a broad scale, cannot provide information about putative smaller populations around the regions contained within the WN stock or finer structure patterns. Nonetheless, comparisons between the North Atlantic and the Pacific Ocean samples, which yielded significant *F_ST_* values, demonstrated that the SNP panel used in this study was capable of separating populations when differences are clear even though it was not designed to differentiate these populations. One final limitation is that samples from the Northwest Atlantic do not extend beyond Baffin Bay. However, the latest research, based on whole‐genome sequencing, does not provide any evidence of structure between Baffin Bay and further south, toward Newfoundland (Ferchaud, Normandeau et al. 2022), suggesting that the entire Northwest Atlantic forms a single genetic cluster.

### 4.2 Effect of Sex‐Associated Loci on the Estimates of Population Structure

We found that including SNPs located on chromosomes 10 and 21, which contain the purported sex‐determining genes (Ferchaud, Mérot et al. 2022), considerably increased the population structure shown by the data. The magnitude of genetic differentiation was greatest when including sex‐associated SNPs and the effect was more pronounced in males than in females. Males appeared to form two distinct groups separated from females in the PCoA when including sex‐associated SNPs and the East–West differentiation was clear. With the removal of sex‐associated SNPs, the structure pattern was much weaker and seemed to follow an isolation‐by‐distance pattern. Since the study relies on a relatively small set of SNPs, one could argue that the removal of even a small number of genetic markers could substantially impact the level of structure observed in terms of statistical power. However, the PCoA pattern using only the sex‐associated markers showed the same pattern as observed in the PCoA using all loci, indicating the strength of these markers in causing the bias. Given that the SNPs used here showed greater genetic differentiation within SNP datasets from previous literature (Westgaard et al. 2017; Gíslason et al. 2023; Berghuis et al. in prep) and that more than 30% were located on chromosomes linked to sex determination, it is very likely that previous population genetic works based on these loci, have unknowingly incorporated the influence of sex differences in their results and interpretations. Notice for this that just a handful of sex‐associated loci can significantly impact genetic structure (Benestan et al. 2017).

After removing the sex‐associated SNPs, we found genetic differences in the North Atlantic follow an isolation‐by‐distance model with two clusters representing the east and west but without complete separation in any of the studied locations. In fact, the SNPs poorly assigned individuals to either cluster, contrasting with the result in Gíslason et al. (2023). Most previous research did not find significant differences within the western NWA or the eastern NEA stocks (Fairbairn 1981; Vis et al. 1997; Knutsen et al. 2007; Roy et al. 2014; Westgaard et al. 2017; Ferchaud, Mérot et al. 2022), and neither did we. However, our results support a gradual turnover of genetic signatures between the two regions in the North Atlantic (Westgaard et al. 2017; Knutsen et al. 2007; Gíslason et al. 2023). This result contrasts with the current stock delineations that recognize four offshore stocks in the North Atlantic and better supports independently derived evidence from tagging experiments (Vihtakari et al. 2022) and survey data (Úbeda et al. 2023), which indicate at most two main biological units of Greenland halibut in the Atlantic but with incomplete isolation. This difference between eastern and western parts of the North Atlantic has been observed in other species such as Atlantic cod (Pogson 2001), haddock (Berg et al. 2021), Atlantic halibut (Foss, Imsland, and Nævdal 1998), lumpfish (Whittaker, Consuegra, and Garcia de Leaniz 2018; Jansson et al. 2023), and beaked redfish (Saha et al. 2016; Saha et al. 2021) among others, and could indicate a shared evolutionary history, and or indicate common drivers of divergence.

### 4.3 Causes for the Inclusion of Sex‐Associated SNPs in the Original Panel

We here discuss two possible non‐exclusive reasons for the inclusion of sex‐associated SNPs in the original design of the SNP panel: first, a difference in the sex ratio among samples, where comparisons are done between samples composed mainly of females and samples composed mainly of males; second, an incomplete evolution of genetic sex determination where linkage with sex phenotypes is heterogenous across the species distribution.

Skewed sex ratios are common in fish species, and they may respond to different factors, like different growth requirements (Klimley 1987), an increased mortality of male embryos (Morán, Labbé, and García de Leaniz 2016), or environmental sex determination (Luckenbach et al. 2009), among others. In Atlantic cod, skewed ratios favoring males are known to occur during spawning season in spawning areas (Morgan and Trippel 1996; Fevolden et al. 2015), and it may result from competition between males in procuring the best places for attracting females. In Greenland halibut there is some evidence indicating that males arrive earlier and stay longer in spawning areas (Albert et al. 2001; Yan et al. 2023), but why this happens is still subject to research. Male‐skewed sex ratios have also been found to be the result of temperature extremes in several flatfish species (Luckenbach et al. 2009). This was studied in Atlantic halibut, but no such influence was found (Hauser and Carvalho 2008). Other than this, and to our knowledge, little peer‐reviewed research has been published exploring naturally occurring sex ratios in the Hippoglossinae subfamily (Greenland, Atlantic and Pacific halibut). Gaining an understanding of this can help on the management of fisheries, ensuring for example that one sex does not get overfished relative to the other, and on the proper design of sampling and experiments.

Sex‐determination systems have been recently studied in three species of the Hippoglossinae subfamily. Both Atlantic and Greenland halibut show evidence of an XY sex‐determination system (Einfeldt et al. 2021; Ferchaud, Mérot et al. 2022) while Pacific halibut shows a ZW (Jasonowicz et al. 2022 and references therein). For Greenland halibut, chromosomes 10 and 21 have been found to harbor the biggest divergence between males and females and based on their evidence, Ferchaud, Mérot et al. (2022) proposed an evolving XY system caused by fusion of autosomes into a Y chromosome. A common feature of evolving sex‐determination systems such as this, is geographic heterogeneity where the genetic sex‐determination system varies across the species' distribution (Kitano and Peichel 2012; Bracewell et al. 2017; Pan et al. 2021). We present here several results that are suggestive of this. First, the sex‐associated loci in this study indicated a pattern of structure where male genotypes varied with longitude. Second, classification errors of visually assigned phenotypes according to the sex‐associated loci varied across the Atlantic regions, with higher error in males than in females. Notice that, given the little divergence on sex‐associated between phenotypic sexes in Svalbard, the worse classification in the eastern locations could have been caused by this location alone. Finally, heterozygosity comparisons between males and females within each location showed loci to split sexes clearly but not in the same locations. Heterozygosity was higher in males than in females downstream of both chromosomes in the eastern samples, while it was upstream in western samples. A possible explanation for this observation may be two inversion polymorphisms, one on each of the sex‐associated chromosomes, affecting males across the Atlantic distribution. These results are not conclusive and call for further exploration of the genomic architecture of sex determination in Greenland halibut across its range.

An exception to the observations on genetic differentiation and heterozygosity differences between sexes is the sample from Svalbard where sex‐associated loci had zero or close to zero values of *F_ST_* and barely any heterozygosity differences between sexes. This could be due to error on the visual identification of sex, where one sex (according to the results on heterozygosity comparisons it would be males) is predominant. Given that individuals in Svalbard are on average the smallest of all samples, this is a likely possibility and emphasizes the need for tools to identify sex genetically (Weise et al. 2023). Nonetheless, in a nursery ground, a sufficiently high number of individuals in a random sample would be assumed to be close to an equal sex ratio. Then, one other reason, for what we observe in Svalbard and following this logic, is that sex determination is not genetic but perhaps environmental. This has also been suggested (but not proven) by Einfeldt et al. (2021) for Atlantic halibut, namely, the simultaneous presence of genetic and environmental sex‐determination mechanisms within the same species. This would then indicate the presence of two sex‐determination systems within the North Atlantic, with an environmentally driven system being more common in the east.

## 5 Conclusions

We found that published discriminatory markers in Greenland halibut have a strong sex‐linked signal, so it is likely that previous research has unknowingly incorporated sex‐biases in their conclusions. We suggest that the incorporation of these SNPs on previous population structure studies is caused by a heterogeneous sex‐determination system across the entire distribution. We base this on the observation that SNPs located in the sex chromosomes 10 and 21 split males in two groups, one in the East and one in the West. For species with a clear sexual dimorphism, disregarding the genetic determination of sex and the different behaviors between sexes, can lead to erroneous understanding of genetic structure. Furthermore, since one of the main steps for successful application of genomics in fisheries management is the creation of SNP panels to discriminate stocks, we also argue that incorporating sex‐biases can cause individuals to be miss‐assigned.

After removal of sex‐associated loci, we still observed a pattern of population structure albeit weaker and consistent with isolation‐by‐distance. Our results better agree with the presence of two different genetic units in the North Atlantic, matching results from tagging data and fisheries models, rather than four stocks, as currently recognized in fisheries management.

Our results call for further effort in taking a whole‐genome approach to characterize the genomic architecture of Greenland halibut across its entire range. This will elucidate the complexity of the sex‐determination system as well as the population structure of this species.

## Author Contributions

**Daniel Estévez‐Barcia:** conceptualization (supporting), data curation (lead), formal analysis (lead), investigation (lead), methodology (lead), resources (equal), software (lead), visualization (lead), writing – original draft (lead), writing – review and editing (equal). **Denis Roy:** conceptualization (supporting), formal analysis (supporting), investigation (supporting), methodology (supporting), resources (equal), supervision (equal), writing – original draft (supporting), writing – review and editing (equal). **Mikko Vihtakari:** conceptualization (supporting), data curation (supporting), formal analysis (supporting), investigation (supporting), methodology (supporting), resources (equal), software (supporting), validation (lead), visualization (supporting), writing – original draft (supporting), writing – review and editing (equal). **Davíð Gíslason:** conceptualization (supporting), investigation (supporting), resources (supporting), writing – review and editing (equal). **Martin Lindegren:** formal analysis (supporting), investigation (supporting), methodology (supporting), software (supporting), visualization (supporting), writing – review and editing (equal). **Asbjørn Christensen:** formal analysis (supporting), methodology (supporting), software (supporting), writing – review and editing (equal). **Margaret Treble:** resources (equal), writing – original draft (supporting), writing – review and editing (equal). **Laura Wheeland:** writing – original draft (supporting), writing – review and editing (equal). **Julio Úbeda:** writing – original draft (supporting), writing – review and editing (equal). **Adriana Nogueira:** writing – original draft (supporting), writing – review and editing (equal). **Kevin Hedges:** writing – original draft (supporting), writing – review and editing (equal). **Áki Jarl Láruson:** validation (equal), writing – original draft (supporting), writing – review and editing (equal). **Alejandro Mateos Rivera:** methodology (equal), resources (equal), writing – original draft (supporting), writing – review and editing (equal). **Geir Dahle:** methodology (equal), resources (equal), writing – review and editing (equal). **Jon‐Ivar Westgaard:** formal analysis (supporting), supervision (supporting), writing – review and editing (equal). **Bjarki Elvarsson:** resources (equal), writing – review and editing (equal). **Lise Helen Ofstad:** resources (equal), writing – review and editing (equal). **Elvar H. Hallfredsson:** supervision (supporting), writing – review and editing (equal). **Ole Thomas Albert:** supervision (supporting), writing – review and editing (equal). **Jesper Boje:** conceptualization (equal), funding acquisition (lead), project administration (lead), supervision (supporting), writing – review and editing (equal). **Torild Johansen:** conceptualization (equal), formal analysis (supporting), methodology (supporting), resources (equal), supervision (lead), writing – original draft (supporting), writing – review and editing (equal).

## Ethics Statement

No animal experiments were performed for the current research. All samples were collected during scientific surveys with congruous bottom‐trawling permissions.

## Conflicts of Interest

The authors declare no conflicts of interest.

## Benefit‐Sharing

The research described here resulted from an international assembly of researchers concerned with sustainable exploitation of marine fisheries. The research process and results have been shared with politicians and stakeholders in Greenland, and among participants in international workshops.

## Supporting information


Data S1.

## Data Availability

Individual genotype data are divided in three datasets: (1) previous to all filters (Gend1), (2) after all relevant filters (Gend2), and (3) after all relevant filters and excluding markers in chromosomes 10 and 21 which include sex‐determination genes. All of these are stored in an R data file as genind lists in Figshare (DOI: 10.6084/m9.figshare.25579806.v2). Sex, length, coordinates, and other relevant metadata, are also stored in Figshare (DOI: 10.6084/m9.figshare.25579815.v2).

## References

[ece370822-bib-0001] Albert, O. T. , Y. Lambert , T. Vollen , C. Freitas , and L. Heggebakken . 2011. “Distinguishing Pelagic and Demersal Swimming of Deepwater Flatfish by Recording of Body Angles.” In Advances in Fish Tagging and Marking Technology. American Fisheries Society Symposium 76, edited by J. McKenzie , B. Parsons , A. Seitz , R. K. Kopf , M. Mesa , and Q. Phelps , 507–528. Bethesda, MD: American Fisheries Society Symposium.

[ece370822-bib-0002] Albert, O. T. , E. M. Nilssen , A. Stene , A. C. Gundersen , and K. H. Nedreaas . 2001. “Maturity Classes and Spawning Behaviour of Greenland Halibut ( *Reinhardtius hippoglossoides* ).” Fisheries Research 51: 217–228. 10.1016/S0165-7836(01)00247-8.

[ece370822-bib-0003] Albert, O. T. , and T. Vollen . 2015. “A Major Nursery Area Around the Svalbard Archipelago Provides Recruits for the Stocks in Both Greenland Halibut Management Areas in the Northeast Atlantic.” ICES Journal of Marine Science 72: 872–879. 10.1093/icesjms/fsu191.

[ece370822-bib-0004] Andersson, L. , D. Bekkevold , F. Berg , et al. 2024. “How Fish Population Genomics Can Promote Sustainable Fisheries: A Road Map.” Annual Review of Animal Biosciences 12: 1–20. 10.1146/annurev-animal-021122-102933. 37906837

[ece370822-bib-0005] Antao, T. , A. Lopes , R. J. Lopes , A. Beja‐Pereira , and G. Luikart . 2008. “LOSITAN: A Workbench to Detect Molecular Adaptation Based on a FST‐Outlier Method.” BMC Bioinformatics 9: 323. 10.1186/1471-2105-9-323. 18662398 PMC2515854

[ece370822-bib-0006] Benestan, L. , J.‐S. S. Moore , B. J. G. G. Sutherland , et al. 2017. “Sex Matters in Massive Parallel Sequencing: Evidence for Biases in Genetic Parameter Estimation and Investigation of Sex Determination Systems.” Molecular Ecology 26: 6767–6783. 10.1111/mec.14217. 28658525

[ece370822-bib-0007] Benjamini, Y. , and Y. Hochberg . 1995. “Controlling the False Discovery Rate: A Practical and Powerful Approach to Multiple Testing. Journal of the Royal Statistical Society.” Series B (Methodological) 57: 289–300. https://www.jstor.org/stable/2346101.

[ece370822-bib-0008] Berg, P. R. , P. E. Jorde , K. A. Glover , et al. 2021. “Genetic Structuring in Atlantic Haddock Contrasts With Current Management Regimes.” ICES Journal of Marine Science 78: 1–13. 10.1093/icesjms/fsaa204.

[ece370822-bib-0009] Berghuis, N. , D. Estevez‐Barcia , M. Vihtakari , et al. (in prep) “Population Genomics and Local Adaptation of Greenland Halibut in the Northwest Atlantic and Western Canadian Arctic.”

[ece370822-bib-0010] Boje, J. , and E. Hjörleifsson . 2000. Nursery Grounds for the West Nordic Greenland Halibut Stock–Where Are They? ICES 2000 Annual Science Conference, Bruges, Belgium. CM 2000/N:03.

[ece370822-bib-0011] Bowering, W. R. , and W. B. Brodie . 1995. Greenland Halibut ( *Reinhardtius hippoglossoides* ). A Review of the Dynamics of Its Distribution and Fisheries Off Eastern Canada and Greenland, edited by A. G. Hopper , 113–160. Dordrecht: Springer Netherlands. 10.1007/978-94-015-84142_4.

[ece370822-bib-0012] Bracewell, R. R. , B. J. Bentz , B. T. Sullivan , and J. M. Good . 2017. “Rapid Neo‐Sex Chromosome Evolution and Incipient Speciation in a Major Forest Pest.” Nature Communications 8: 1593. 10.1038/s41467-017-01761-4. PMC5693900 29150608

[ece370822-bib-0013] Bradbury, I. R. , S. Hubert , B. Higgins , et al. 2010. “Parallel Adaptive Evolution of Atlantic Cod on Both Sides of the Atlantic Ocean in Response to Temperature.” Proceedings of the Royal Society B: Biological Sciences 277: 3725–3734. 10.1098/rspb.2010.0985. PMC2992707 20591865

[ece370822-bib-0014] Breiman, L. 2001. “Random Forests.” Machine Learning 45: 5–32. 10.1023/A:1010933404324.

[ece370822-bib-0015] Camacho, C. , G. Coulouris , V. Avagyan , et al. 2009. “BLAST+: Architecture and Applications.” BMC Bioinformatics 10: 421. 20003500 10.1186/1471-2105-10-421 PMC2803857

[ece370822-bib-0016] Carrier, E. , A.‐L. Ferchaud , E. Normandeau , P. Sirois , and L. Bernatchez . 2020. “Estimating the Contribution of Greenland Halibut ( *Reinhardtius hippoglossoides* ) Stocks to Nurseries by Means of Genotyping‐By‐Sequencing: Sex and Time Matter.” Evolutionary Applications 13: 2155–2167. 10.1111/eva.12979. 33005216 PMC7513701

[ece370822-bib-0017] Domínguez‐Petit, R. , P. Ouellet , and Y. Lambert . 2013. “Reproductive Strategy, Egg Characteristics and Embryonic Development of Greenland Halibut ( *Reinhardtius hippoglossoides* ).” ICES Journal of Marine Science 70: 342–351. 10.1093/icesjms/fss180.

[ece370822-bib-0018] Dray, S. , and A.‐B. Dufour . 2007. “The ade4 Package: Implementing the Duality Diagram for Ecologists.” Journal of Statistical Software 22, no. 4: 1–20. 10.18637/jss.v022.i04.

[ece370822-bib-0019] Dwyer, K. S. S. , A. Buren , and M. Koen‐Alonso . 2010. “Greenland Halibut Diet in the Northwest Atlantic From 1978 to 2003 as an Indicator of Ecosystem Change.” Journal of Sea Research 64: 436–445. 10.1016/j.seares.2010.04.006.

[ece370822-bib-0020] Earl, D. A. , and B. M. vonHoldt . 2012. “STRUCTURE HARVESTER: A Website and Program for Visualizing STRUCTURE Output and Implementing the Evanno Method.” Conservation Genetics Resources 4: 359–361. 10.1007/s12686-011-9548-7.

[ece370822-bib-0021] Einfeldt, A. L. , T. Kess , A. Messmer , et al. 2021. “Chromosome Level Reference of Atlantic Halibut *Hippoglossus hippoglossus* Provides Insight Into the Evolution of Sexual Determination Systems.” Molecular Ecology Resources 21: 1686–1696. 33655659 10.1111/1755-0998.13369

[ece370822-bib-0022] Evanno, G. , S. Regnaut , and J. Goudet . 2005. “Detecting the Number of Clusters of Individuals Using the Software STRUCTURE: A Simulation Study.” Molecular Ecology 14: 2611–2620. 10.1111/j.1365-294X.2005.02553.x. 15969739

[ece370822-bib-0023] Fairbairn, D. J. 1981. “Biochemical Genetic Analysis of Population Differentiation in Greenland Halibut ( *Reinhardtius hippoglossoides* ) From the Northwest Atlantic, Gulf of St. Lawrence, and Bering Sea.” Canadian Journal of Fisheries and Aquatic Sciences 38: 669–677. 10.1139/f81-090.

[ece370822-bib-0024] Ferchaud, A.‐L. , C. Mérot , E. Normandeau , et al. 2022. “Chromosome‐Level Assembly Reveals a Putative y‐Autosomal Fusion in the Sex Determination System of the Greenland Halibut ( *Reinhardtius hippoglossoides* ).” G3: Genes, Genomes, Genetics 12: jkab376. 10.1093/g3journal/jkab376. 34791178 PMC8727965

[ece370822-bib-0025] Ferchaud, A.‐L. , E. Normandeau , C. Babin , et al. 2022. “A Cold‐Water Fish Striving in a Warming Ocean: Insights From Whole‐Genome Sequencing of the Greenland Halibut in the Northwest Atlantic.” Frontiers in Marine Science 9: 992504. 10.3389/fmars.2022.992504.

[ece370822-bib-0095] Fevolden, S. , J. Westgaard , and T. Pedersen . 2015. “Extreme Male‐Skewed Sex Ratios on Spawning Grounds for Atlantic Cod *Gadus morhua* with Typical Coastal Cod Signatures of the Pan I (Pantophysin) Locus.” Sexuality and Early Development in Aquatic Organisms 1, no. 2: 133–142. 10.3354/sedao00013.

[ece370822-bib-0026] Foll, M. , and O. Gaggiotti . 2008. “A Genome‐Scan Method to Identify Selected Loci Appropriate for Both Dominant and Codominant Markers: A Bayesian Perspective.” Genetics 180: 977–993. 10.1534/genetics.108.092221. 18780740 PMC2567396

[ece370822-bib-0027] Foss, A. , A. K. Imsland , and G. Nævdal . 1998. “Population Genetic Studies of the Atlantic Halibut in the North Atlantic Ocean.” Journal of Fish Biology 53: 901–905. 10.1111/j.1095-8649.1998.tb01845.x.

[ece370822-bib-0028] Fraser, D. J. , and L. Bernatchez . 2001. “Adaptive Evolutionary Conservation: Towards a Unified Concept Fordefining Conservation Units.” Molecular Ecology 10: 2741–2752. 10.1046/j.0962-1083.2001.01411.x. 11903888

[ece370822-bib-0029] Fuentes‐Pardo, A. P. , E. D. Farrell , M. E. Pettersson , C. G. Sprehn , and L. Andersson . 2023. “The Genomic Basis and Environmental Correlates of Local Adaptation in the Atlantic Horse Mackerel ( *Trachurus trachurus* ).” Evolutionary Applications 16: 1201–1219. 10.1111/eva.13559. 37360028 PMC10286234

[ece370822-bib-0030] Fuentes‐Pardo, A. P. , R. Stanley , C. Bourne , et al. 2024. “Adaptation to Seasonal Reproduction and Environment‐Associated Factors Drive Temporal and Spatial Differentiation in Northwest Atlantic Herring Despite Gene Flow.” Evolutionary Applications 2024, no. 17: e13675. 10.1111/eva.13675 PMC10940790 38495946

[ece370822-bib-0031] Gagnaire, P.‐A. , T. Broquet , D. Aurelle , et al. 2015. “Using Neutral, Selected, and Hitchhiker Loci to Assess Connectivity of Marine Populations in the Genomic Era.” Evolutionary Applications 8: 769–786. 10.1111/eva.12288. 26366195 PMC4561567

[ece370822-bib-0032] Gíslason, D. , D. Estévez‐Barcia , S. Sveinsson , et al. 2023. “Population Structure Discovered in Juveniles of Greenland Halibut ( *Reinhardtius hippoglossoides* Walbaum, 1792).” ICES Journal of Marine Science 80: 889–896. 10.1093/icesjms/fsad011.

[ece370822-bib-0033] Godo, O. R. , and T. Haug . 1989. “A Review of the Natural History, Fisheries, and Management of Greenland Halibut ( *Reinhardtius hippoglossoides* ) in the Eastern Norwegian and Barents Seas.” ICES Journal of Marine Science 46: 62–75. 10.1093/icesjms/46.1.62.

[ece370822-bib-0034] Goudet, J. , and T. Jombart . 2022. “Hierfstat: Estimation and Tests of Hierarchical f‐Statistics.” https://CRAN.R‐project.org/package=hierfstat.

[ece370822-bib-0035] Gundersen, A. C. C. , J. Kennedy , A. Woll , I. Fossen , and J. Boje . 2013. “Identifying Potential Greenland Halibut Spawning Areas and Nursery Grounds Off East and South‐Western Greenland and Its Management Implications.” Journal of Sea Research 75: 110–117. 10.1016/j.seares.2012.05.016.

[ece370822-bib-0036] Hauser, L. , and G. Carvalho . 2008. “Paradigm Shifts in Marine Fisheries Genetics: Ugly Hypotheses Slain by Beautiful Facts.” Fish and Fisheries 9: 333–362.

[ece370822-bib-0037] Höglund, J. 2009. Evolutionary Conservation Genetics. Oxford University Press. Oxford. https://academic.oup.com/book/12619.

[ece370822-bib-0038] Hovde, S. C. , O. T. Albert , and E. M. Nilssen . 2002. “Spatial, Seasonal and Ontogenetic Variation in Diet of Northeast Arctic Greenland Halibut ( *Reinhardtius hippoglossoides* ).” ICES Journal of Marine Science 59: 421–437. 10.1006/jmsc.2002.1171.

[ece370822-bib-0039] Igland, O. T. , and G. Nævdal . 2001. “Allozyme Studies of Greenland Halibut, *Reinhardtius hippoglossoides* Walbaum 1792, From the North Atlantic.” Sarsia 86: 237–240. 10.1080/00364827.2001.10420480.

[ece370822-bib-0040] Jakobsson, M. , and N. A. Rosenberg . 2007. “CLUMPP: A Cluster Matching and Permutation Program for Dealing With Label Switching and Multimodality in Analysis of Population Structure.” Bioinformatics 23: 1801–1806. 10.1093/bioinformatics/btm233. 17485429

[ece370822-bib-0041] Jansson, E. , E. Faust , D. Bekkevold , et al. 2023. “Global, Regional, and Cryptic Population Structure in a High Gene‐Flow Transatlantic Fish.” PLoS One 18: e0283351. 10.1371/journal.pone.0283351. 36940210 PMC10027230

[ece370822-bib-0042] Jasonowicz, A. J. , A. Simeon , M. Zahm , et al. 2022. “Generation of a Chromosome‐Level Genome Assembly for Pacific Halibut (*Hippoglossus stenolepsis*) and Characterization of Its Sex‐Determining Genomic Region.” Molecular Ecology Resources 2022: 1–16. 10.1111/1755-0998.13641 PMC9541706 35569134

[ece370822-bib-0043] Jensen, A. S. 1935. The Greenland Halibut ( *Reinhardtius hippoglossoides* (Walb)) its Development and Migra‐ Tions. Copenhagen: Levin & Munksgaard.

[ece370822-bib-0044] Jørgensen, O. A. 1997. “Movement Patterns of Greenland Halibut, *Reinhardtius hippoglossoides* (Walbaum), at West Greenland, as Inferred From Trawl Survey Distribution and Size Data.” Journal of Northwest Atlantic Fishery Science 21: 23–37. 10.2960/J.v21.a2.

[ece370822-bib-0045] Junquera, S. , and J. Zamarro . 1994. “Sexual Maturity and Spawning of Greenland Halibut ( *Reinhardtius hippoglossoides* ) From Flemish Pass Area.” NAFO Scientific Council Studies 20: 47–52.

[ece370822-bib-0046] Kamvar, Z. N. , J. F. Tabima , and N. J. Grünwald . 2014. “Poppr: An R Package for Genetic Analysis of Populations With Clonal, Partially Clonal, and/or Sexual Reproduction.” PeerJ 2: e281. 10.7717/peerj.281. 24688859 PMC3961149

[ece370822-bib-0047] Kitano, J. , and C. L. Peichel . 2012. “Turnover of Sex Chromosomes and Speciation in Fishes.” Environmental Biology of Fishes 94: 549–558. 10.1007/s10641-011-9853-8. 26069393 PMC4459657

[ece370822-bib-0048] Klimley, P. A. 1987. “The Determinants of Sexual Segregation in the Scalloped Hammerhead Shark, *Sphyrna lewini* .” Environmental Biology of Fishes 18: 27–40. 10.1007/BF00002325.

[ece370822-bib-0049] Knutsen, H. , P. E. Jorde , O. T. Albert , A. R. Hoelzel , and N. C. Stenseth . 2007. “Population Genetic Structure in the North Atlantic Greenland Halibut ( *Reinhardtius hippoglossoides* ): Influenced by Oceanic Current Systems?” Canadian Journal of Fisheries and Aquatic Sciences 64: 857–866.

[ece370822-bib-0050] Liaw, A. , and M. Wiener . 2002. “Classification and Regression by Random Forest.” 2, 18–22. https://CRAN.R‐project.org/doc/Rnews/.

[ece370822-bib-0051] Luckenbach, J. A. , R. J. Borski , H. V. Daniels , and J. Godwin . 2009. “Sex Determination in Flatfishes: Mechanisms and Environmental Influences.” Seminars in Cell and Developmental Biology 20: 256–263. 10.1016/j.semcdb.2008.12.002. 19121404

[ece370822-bib-0052] Luu, K. , E. Bazin , and M. G. B. Blum . 2017. “Pcadapt: An r Package to Perform Genome Scans for Selection Based on Principal Component Analysis.” Molecular Ecology Resources 17: 67–77. 10.1111/1755-0998.12592. 27601374

[ece370822-bib-0053] Magnússon, J. V. 1977. “Notes on the Eggs and Larvae of Greenland Halibut at Iceland.” ICES C.M., 1977, 6.

[ece370822-bib-0054] Mariani, S. , W. F. Hutchinson , E. M. C. Hatfield , et al. 2005. “North Sea Herring Population Structure Revealed by Microsatellite Analysis.” Marine Ecology Progress Series 303: 245–257. 10.3354/meps303245.

[ece370822-bib-0055] Miller, J. M. , C. I. Cullingham , and R. M. Peery . 2020. “The Influence of a Priori Grouping on Inference of Genetic Clusters: Simulation Study and Literature Review of the DAPC Method.” Heredity 125: 269–280. 10.1038/s41437-020-0348-2. 32753664 PMC7553915

[ece370822-bib-0056] Morán, P. , L. Labbé , and C. García de Leaniz . 2016. “The Male Handicap: Male‐Biased Mortality Explains Skewed Sex Ratios in Brown Trout Embryos.” Biology Letters 12: 20160693. 10.1098/rsbl.2016.0693. 27928001 PMC5206587

[ece370822-bib-0096] Morgan, M. 1996. “Skewed Sex Ratios in Spawning Shoals of Atlantic Cod (*Gadus morhua*).” ICES Journal of Marine Science 53, no. 5: 820–826. 10.1006/jmsc.1996.0103.

[ece370822-bib-0057] Nielsen, E. E. , J. Hemmer‐Hansen , P. F. Larsen , and D. Bekkevold . 2009. “Population Genomics of Marine Fishes: Identifying Adaptive Variation in Space and Time.” Molecular Ecology 18: 3128–3150. 19627488 10.1111/j.1365-294X.2009.04272.x

[ece370822-bib-0058] Nogueira, A. , X. Paz , and D. González‐Troncoso . 2017. “Demersal Groundfish Assemblages and Depth‐ Related Trends on Flemish Cap (NAFO Division 3M): 2004–2013.” Fisheries Research 186: 192–204. 10.1016/j.fishres.2016.08.016.

[ece370822-bib-0059] Orlova, S. Y. , A. A. Volkov , D. M. Shcepetov , et al. 2019. “Inter‐ and Intra‐Species Relationships of Greenland Halibut *Reinhardtius hippoglossoides* (Pleuronectidae) Based on the Analysis of Nuclear and Mitochondrial Genetic Markers.” Journal of Ichthyology 59: 65–77. 10.1134/S0032945219010119.

[ece370822-bib-0060] Orlova, S. Y. , A. A. Volkov , O. A. Maznikova , N. V. Chernova , I. I. Glebov , and A. M. Orlov . 2017. “Population Status of Greenland Halibut *Reinhardtius hippoglossoides* (Walbaum, 1793) of the Laptev Sea.” Doklady Biochemistry and Biophysics 477: 349–353. 10.1134/S1607672917060023. 29297122

[ece370822-bib-0061] Ovenden, J. R. , O. Berry , D. J. Welch , R. C. Buckworth , and C. M. Dichmont . 2015. “Ocean's Eleven: A Critical Evaluation of the Role of Population, Evolutionary and Molecular Genetics in the Management of Wild Fisheries.” Fish and Fisheries 16: 125–159. 10.1111/faf.12052.

[ece370822-bib-0062] Pan, Q. , R. Feron , E. Jouanno , et al. 2021. “The Rise and Fall of the Ancient Northern Pike Master Sex‐Determining Gene.” eLife 10: e62858. 10.7554/eLife.62858. 33506762 PMC7870143

[ece370822-bib-0063] Pogson, G. H. 2001. “Nucleotide Polymorphism and Natural Selection at the Pantophysin (Pan I) Locus in the Atlantic Cod, *Gadus morhua* (L.).” Genetics 157: 317–330. 10.1093/genetics/157.1.317. 11139512 PMC1461473

[ece370822-bib-0064] Pomilla, C. , M. A. Treble , L. D. Postma , M. M. Lindsay , and J. D. Reist . 2008. “Initial Genetic Evidence of Population Structure of Greenland Halibut ( *Reinhardtius hippoglossoides* ) in the Northwest Atlantic.” Journal of Northwest Atlantic Fisheries Sciences 40: 1–15. 10.2960/J.v40.m637.

[ece370822-bib-0065] Pritchard, J. K. , M. Stephens , and P. Donnelly . 2000. “Inference of Population Structure Using Multilocus Genotype Data.” Genetics 155: 945–959. 10.1093/genetics/155.2.945. 10835412 PMC1461096

[ece370822-bib-0066] R Core Team . 2022. “R: A Language and Environment for Statistical Computing.” https://www.R‐project.org/.

[ece370822-bib-0067] Riget, F. , and J. Boje . 1988. “Distribution and Abundance of Young Greenland Halibut ( *Reinhardtius hippoglossoides* ) in West Greenland Waters.” NAFO Science Council Studies 12: 7–12.

[ece370822-bib-0068] Riget, F. , J. Boje , and V. Simonsen . 1992. “Analysis of Meristic Characters and Genetic Differentiation in Greenland Halibut ( *Reinhardtius hippoglossoides* ) in the Northwest Atlantic.” Journal of Northwest Atlantic Fishery Science 12: 7–14. 10.2960/J.v12.a1.

[ece370822-bib-0069] Rousset, F. 2008. “GENEPOP'007: A Complete Re‐Implementation of the GENEPOP Software for Windows and Linux.” Molecular Ecology Resources 8: 103–106. 10.1111/j.1471-8286.2007.01931.x. 21585727

[ece370822-bib-0070] Roy, D. , D. C. Hardie , M. A. Treble , J. D. Reist , and D. E. Ruzzante . 2014. “Evidence Supporting Panmixia in Greenland Halibut ( *Reinhardtius hippoglossoides* ) in the Northwest Atlantic.” Canadian Journal of Fisheries and Aquatic Sciences 71: 763–774. 10.1139/cjfas-2014-0004.

[ece370822-bib-0071] Saha, A. , T. Johansen , R. Hedeholm , et al. 2016. “Geographic Extent of Introgression in Sebastes Mentella and Its Effect on Genetic Population Structure.” Evolutionary Applications 10: 77–90. 10.1111/eva.12429. 28035237 PMC5192944

[ece370822-bib-0072] Saha, A. , M. Kent , L. Hauser , et al. 2021. “Hierarchical Genetic Structure in an Evolving Species Complex: Insights From Genome Wide ddRAD Data in *Sebastes mentella* .” PLoS One 16: e0251976. 10.1371/journal.pone.0251976. 34043665 PMC8158871

[ece370822-bib-0073] Sohn, D. , L. Ciannelli , and J. T. Duffy‐Anderson . 2010. “Distribution and Drift Pathways of Greenland Halibut ( *Reinhardtius hippoglossoides* ) During Early Life Stages in the Eastern Bering Sea and Aleutian Islands.” Fisheries Oceanography 19: 339–353. 10.1111/j.1365-2419.2010.00549.x.

[ece370822-bib-0074] Solmundsson, J. 2007. “Trophic Ecology of Greenland Halibut ( *Reinhardtius hippoglossoides* ) on the Icelandic Continental Shelf and Slope.” Marine Biology Research 3: 231–242. 10.1080/17451000701477513.

[ece370822-bib-0075] Torgerson, W. S. 1958. Theory and Methods of Scaling. New York: John Wiley and Sons, Inc.

[ece370822-bib-0076] Tremblay‐Gagnon, F. , S. Brown‐Vuillemin , K. Skanes , et al. 2023. “Spatiotemporal Variability in Diet Composition of Greenland Halibut ( *Reinhardtius hippoglossoides* ) From the Eastern Canadian Arctic.” Journal of Fish Biology 103: 1430–1444. 10.1111/jfb.15519. 37563757

[ece370822-bib-0077] Úbeda, J. , A. Nogueira , N. Tolimieri , et al. 2023. “Using Multivariate Autoregressive State‐Space Models to Examine Stock Structure of Greenland Halibut in the North Atlantic.” Fisheries Management and Ecology 30: 521–535. 10.1111/fme.12639.

[ece370822-bib-0078] van Wyngaarden, M. , P. V. R. Snelgrove , C. DiBacco , et al. 2017. “Identifying Patterns of Dispersal, Connectivity and Selection in the Sea Scallop, *Placopecten magellanicus* , Using RADseq‐Derived SNPs.” Evolutionary Applications 10: 102–117. 28035239 10.1111/eva.12432 PMC5192885

[ece370822-bib-0079] Vihtakari, M. , B. Þ. Elvarsson , M. Treble , et al. 2022. “Migration Patterns of Greenland Halibut in the North Atlantic Revealed by a Compiled Mark–Recapture Dataset.” ICES Journal of Marine Science 79: 1902–1917. 10.1093/icesjms/fsac127.

[ece370822-bib-0080] Vihtakari, M. , R. Hordoir , M. Treble , et al. 2021. “Pan‐Arctic Suitable Habitat Model for Greenland Halibut.” ICES Journal of Marine Science 78: 1340–1356. 10.1093/icesjms/fsab007.

[ece370822-bib-0081] Vis, M. L. , S. M. Carr , W. R. Bowering , and W. S. Davidson . 1997. “Greenland Halibut ( *Reinhardtius hippoglossoides* ) in the North Atlantic Are Genetically Homogeneous.” Canadian Journal of Fisheries and Aquatic Sciences 54: 1813–1821. 10.1139/f97-088.

[ece370822-bib-0082] Wang, J. , and L. S. Chen . 2016. “MixRF: A Random‐Forest‐Based Approach for Imputing Clustered Incomplete Data.” https://CRAN.R‐project.org/package=MixRF.

[ece370822-bib-0083] Waples, R. S. 1998. “Separating the Wheat From the Chaff: Patterns of Genetic Differentiation in High Gene Flow Species.” Journal of Heredity 89: 438–450. 10.1093/jhered/89.5.438.

[ece370822-bib-0084] Weir, B. S. , and C. C. Cockerham . 1984. “Estimating f‐Statistics for the Analysis of Population Structure.” Evolution 38: 1358. 28563791 10.1111/j.1558-5646.1984.tb05657.x

[ece370822-bib-0085] Weise, E. M. , M. van Wyngaarden , C. Den Heyer , et al. 2023. “SNP Panel and Genomic Sex Identification in Atlantic Halibut ( *Hippoglossus hippoglossus* ).” Marine Biotechnology 25: 580–587. 37351707 10.1007/s10126-023-10227-2

[ece370822-bib-0086] Westgaard, J.‐I. , A. Saha , M. Kent , et al. 2017. “Genetic Population Structure in Greenland Halibut ( *Reinhardtius hippoglossoides* ) and Its Relevance to Fishery Management.” Canadian Journal of Fisheries and Aquatic Sciences 74: 475–485. 10.1139/cjfas-2015-0430.

[ece370822-bib-0087] Wheeland, L. J. , and M. J. Morgan . 2020. “Age‐Specific Shifts in Greenland Halibut ( *Reinhardtius hippoglossoides* ) Distribution in Response to Changing Ocean Climate.” ICES Journal of Marine Science 77: 230–240. 10.1093/icesjms/fsz152.

[ece370822-bib-0088] Whittaker, B. A. , S. Consuegra , and C. Garcia de Leaniz . 2018. “Genetic and Phenotypic Differentiation of Lumpfish ( *Cyclopterus lumpus* ) Across the North Atlantic: Implications for Conservation and Aquaculture.” PeerJ 6: e5974. 10.7717/peerj.5974. 30498640 PMC6251346

[ece370822-bib-0089] Wojtasik, B. , A. Kijewska , M. Mioduchowska , B. Mikuła , and J. Sell . 2021. “Temporal Isolation Between Two Strongly Differentiated Stocks of the Greenland Halibut ( *Reinhardtius hippoglossoides* Walbaum, 1792) From the Western Barents Sea.” Polish Polar Research 42: 117–138. 10.24425/ppr.2021.136603.

[ece370822-bib-0090] Wood, S. N. 2011. “Fast Stable Restricted Maximum Likelihood and Marginal Likelihood Estimation of Semiparametric Generalized Linear Models.” Journal of the Royal Statistical Society, Series B: Statistical Methodology 73: 3–36. 10.1111/j.1467-9868.2010.00749.x.

[ece370822-bib-0091] Wood, S. N. 2017. Generalized Additive Models: An Introduction With r. 2nd ed. New York: Chapman; Hall/CRC.

[ece370822-bib-0092] Yan, Y. , E. Cantoni , C. Field , et al. 2023. “Spatio‐Temporal Modelling of Greenland Halibut Maturation Across the Northwest Atlantic.” ICES Journal of Marine Science 2023: 1–15.

[ece370822-bib-0093] Estévez‐Barcia, D. , D. Roy , M. Vihtakari , et al. 2024a. “Metadata. Figshare. Dataset.” 10.6084/m9.figshare.25579815.v2.

[ece370822-bib-0094] Estévez‐Barcia, D. , D. Roy , M. Vihtakari , et al. 2024b. “Genotypes_RDAfile. Figshare. Dataset.” 10.6084/m9.figshare.25579806.v2.

