# Supplementary material for: Sex Influences the Genetic Structure of Greenland Halibut in the North Atlantic
Source: Ecol Evol. 2025 Feb 10;15(2):e70822. doi: 10.1002/ece3.70822 (PMC11810528; doi:10.1002/ece3.70822)
Supplement: Supplementary file 1 — Data S1. [file ECE3-15-e70822-s001.docx]

**Supplemental Information for:**

**Sex influence on the genetic structure of Greenland halibut in the North Atlantic**

Daniel Estévez-Barcia^1*^, Denis Roy^2^, Mikko Vihtakari^3^, Davíð Gíslason^4^, Martin Lindegren^5^, Asbjørn Christensen^5^, Margaret Treble^6^, Laura Wheeland^6^, Julio Úbeda^1^, Adriana Nogueira^1^, Kevin Hedges^6^, Áki Jarl Láruson^7^, Alejandro Mateos Rivera^3^, Geir Dahle^3^, Jon-Ivar Westgaard^3^, Bjarki Elvarsson^7^, Lise Helen Ofstad^8^, Elvar H. Hallfredsson^3^, Ole Thomas Albert^3^, Jesper Boje^1,5^, Torild Johansen^3^

^1^Greenland Institute of Natural Resources, Kivioq 2, 3900, Nuuk, Greenland.

^2^Department of Natural Resource Sciences, McGill University, Lakeshore Rue, Ste-Anne-de-Bellevue, QC H9X 3V9, Montreal, Canada.

^3^Institute of Marine Research, Tromsø department, Fram Centre, Hjalmar Johansensgt. 14, 9007 Tromsø

^4^Matis ltd., Vínlandsleið 12 113, Reykjavík, Iceland.

^5^DTU Aqua, Department of Aquatic Resources. Henrik Dams Allé, Building 201, 2800 Lyngby, Denmark.

^6^Fisheries and Oceans Canada, Freshwater Institute, 501 University Avenue, MB R3T 2N6, Winnipeg, Canada.

^7^Marine and Freshwater Research Institute, Fornubúðum 5, 220 Hafnarfjörður, Iceland.

^8^Faroe Marine Research Institute, Nóatún 1, FO 100, Tórshavn, Faroe Islands.

^*^Corresponding author

**Table of Contents:**

| **Table S1** | Page 2-3 |
| --- | --- |
| **Table S2** | Page 4 |
| **Table S3** | Page 5-6 |
| **Table S4** | Page 7 |
| **Table S5** | Page 7 |
| **Table S6** | Page 8 |
| **Figure S1** | Page 8 |
| **Figure S2** | Page 9-12 |
| **Figure S3** | Page 12 |
| **Figure S4** | Page 13 |
| **Figure S5** | Page 13 |
| **Figure S6** | Page 14 |

**Table S1**. Basic genetic statistics for each locus genotyped for Greenland halibut sampled in the North Atlantic. Observed (H_O_), expected (H_S_) and total (H_T_) heterozygosity are indicated, as well as F_ST_ and F_IS_.

| **Locus** | **H_O_** | **H_S_** | **H_T_** | **F_ST_** | **F_IS_** |
| --- | --- | --- | --- | --- | --- |
| GrHa01 | 0.45 | 0.44 | 0.45 | 0.03 | -0.03 |
| GrHa02 | 0.43 | 0.39 | 0.43 | 0.1 | -0.1 |
| GrHa03 | 0.31 | 0.33 | 0.34 | 0.02 | 0.06 |
| GrHa04 | 0.18 | 0.15 | 0.16 | 0.09 | -0.2 |
| GrHa05 | 0.33 | 0.36 | 0.37 | 0.03 | 0.07 |
| GrHa08 | 0.37 | 0.45 | 0.44 | -0.01 | 0.18 |
| GrHa09 | 0.31 | 0.29 | 0.3 | 0.02 | -0.04 |
| GrHa10 | 0.31 | 0.31 | 0.34 | 0.1 | 0 |
| GrHa11 | 0.11 | 0.09 | 0.1 | 0.08 | -0.15 |
| GrHa12 | 0.15 | 0.17 | 0.17 | 0.01 | 0.13 |
| GrHa13 | 0.48 | 0.5 | 0.5 | 0 | 0.05 |
| GrHa14 | 0.48 | 0.47 | 0.5 | 0.06 | -0.02 |
| GrHa15 | 0.14 | 0.12 | 0.13 | 0.07 | -0.16 |
| GrHa16 | 0.35 | 0.39 | 0.39 | 0 | 0.1 |
| locus01 | 0.38 | 0.37 | 0.38 | 0.01 | -0.03 |
| locus02 | 0.44 | 0.42 | 0.47 | 0.11 | -0.04 |
| locus05 | 0.28 | 0.26 | 0.26 | 0.03 | -0.1 |
| locus06 | 0.09 | 0.09 | 0.08 | 0 | -0.03 |
| locus07 | 0.12 | 0.12 | 0.13 | 0.04 | 0 |
| locus08 | 0.08 | 0.09 | 0.09 | 0.01 | 0.13 |
| locus09 | 0.38 | 0.37 | 0.38 | 0.02 | -0.02 |
| locus11 | 0.54 | 0.5 | 0.5 | 0 | -0.08 |
| locus12 | 0.11 | 0.14 | 0.14 | 0 | 0.21 |
| locus13 | 0.16 | 0.15 | 0.15 | 0 | -0.04 |
| locus14 | 0.34 | 0.38 | 0.38 | 0.01 | 0.1 |
| locus15 | 0.26 | 0.26 | 0.26 | 0 | -0.02 |
| locus17 | 0.34 | 0.38 | 0.38 | 0.01 | 0.1 |
| locus19 | 0.46 | 0.48 | 0.5 | 0.05 | 0.04 |
| locus20 | 0.36 | 0.34 | 0.34 | 0 | -0.06 |
| locus22 | 0.16 | 0.16 | 0.17 | 0.06 | -0.01 |
| locus23 | 0.4 | 0.44 | 0.44 | 0.01 | 0.09 |
| locus26 | 0.25 | 0.24 | 0.24 | 0 | -0.04 |
| locus27 | 0.43 | 0.44 | 0.44 | 0 | 0.03 |
| locus30 | 0.19 | 0.2 | 0.2 | 0 | 0.02 |
| locus31 | 0.47 | 0.48 | 0.49 | 0.02 | 0.01 |
| locus32 | 0.27 | 0.24 | 0.25 | 0.04 | -0.13 |
| locus33 | 0.15 | 0.15 | 0.15 | 0.01 | 0 |
| locus34 | 0.32 | 0.27 | 0.28 | 0.02 | -0.18 |
| locus35 | 0.25 | 0.25 | 0.25 | 0.01 | -0.02 |
| locus36 | 0.49 | 0.42 | 0.43 | 0.01 | -0.15 |

**Table S1 (continued)**. Basic genetic statistics for each locus genotyped for Greenland halibut sampled in the North Atlantic. Observed (H_O_), expected (H_S_) and total (H_T_) heterozygosity are indicated, as well as F_ST_ and F_IS_.

| locus37 | 0.38 | 0.38 | 0.4 | 0.04 | 0.01 |
| --- | --- | --- | --- | --- | --- |
| locus40 | 0.33 | 0.31 | 0.31 | 0.01 | -0.06 |
| locus41 | 0.2 | 0.2 | 0.2 | 0.01 | -0.02 |
| locus43 | 0.46 | 0.49 | 0.5 | 0.02 | 0.06 |
| locus45 | 0.41 | 0.42 | 0.43 | 0.03 | 0.02 |
| locus46 | 0.38 | 0.35 | 0.36 | 0.02 | -0.07 |
| locus47 | 0.43 | 0.44 | 0.44 | 0.02 | 0.01 |
| locus48 | 0.23 | 0.22 | 0.22 | 0.01 | -0.02 |
| locus50 | 0.13 | 0.14 | 0.14 | 0.01 | 0.07 |
| locus51 | 0.12 | 0.14 | 0.15 | 0.09 | 0.14 |
| locus53 | 0.15 | 0.14 | 0.15 | 0.05 | -0.06 |
| locus54 | 0.11 | 0.11 | 0.11 | 0.01 | 0 |
| locus56 | 0.17 | 0.17 | 0.17 | 0.01 | 0 |
| locus58 | 0.21 | 0.2 | 0.21 | 0.02 | -0.05 |
| locus59 | 0.2 | 0.21 | 0.21 | 0.01 | 0.06 |
| locus60 | 0.47 | 0.46 | 0.47 | 0.02 | -0.02 |
| locus61 | 0.28 | 0.28 | 0.32 | 0.14 | -0.01 |
| locus62 | 0.45 | 0.44 | 0.44 | 0 | -0.02 |
| locus63 | 0.14 | 0.13 | 0.14 | 0.04 | -0.04 |
| locus64 | 0.4 | 0.37 | 0.38 | 0.03 | -0.09 |
| locus66 | 0.34 | 0.36 | 0.37 | 0.03 | 0.05 |
| locus67 | 0.26 | 0.26 | 0.28 | 0.09 | -0.01 |
| locus68 | 0.28 | 0.27 | 0.28 | 0.03 | -0.04 |
| locus73 | 0.34 | 0.33 | 0.33 | 0.02 | -0.05 |
| locus74 | 0.44 | 0.45 | 0.47 | 0.03 | 0.01 |
| locus75 | 0.13 | 0.15 | 0.16 | 0.06 | 0.17 |
| locus76 | 0.16 | 0.14 | 0.15 | 0.09 | -0.2 |
| locus78 | 0.13 | 0.13 | 0.13 | 0 | -0.04 |
| locus79 | 0.54 | 0.45 | 0.49 | 0.07 | -0.2 |
| locus80 | 0.23 | 0.23 | 0.23 | 0.01 | 0 |
| locus81 | 0.42 | 0.44 | 0.45 | 0.01 | 0.04 |
| locus82 | 0.48 | 0.43 | 0.43 | 0 | -0.12 |
| locus83 | 0.4 | 0.45 | 0.45 | 0 | 0.11 |
| locus84 | 0.5 | 0.48 | 0.5 | 0.03 | -0.03 |
| locus85 | 0.49 | 0.45 | 0.46 | 0.02 | -0.08 |
| locus86 | 0.14 | 0.14 | 0.14 | 0.03 | -0.01 |
| locus88 | 0.38 | 0.39 | 0.39 | 0 | 0.04 |
| locus89 | 0.45 | 0.43 | 0.43 | 0 | -0.04 |
| locus90 | 0.21 | 0.26 | 0.27 | 0.03 | 0.21 |
| **Overall** | **0.3** | **0.3** | **0.31** | **0.03** | **-0.01** |

**Table S2**. Basic genetic stats for each location genotyped for Greenland halibut. Observed (H_O_) and expected (H_S_) heterozygosity as well as the inbreeding coefficient (F_IS_) are indicated as columns.

| **Location** | **H_O_** | **H_S_** | **F_IS_** |
| --- | --- | --- | --- |
| Pac | 0.24 | 0.23 | -0.024 |
| GrlNW | 0.31 | 0.28 | -0.065 |
| Disko | 0.33 | 0.32 | -0.032 |
| CanNE | 0.33 | 0.32 | -0.04 |
| Davis | 0.33 | 0.31 | -0.054 |
| GrlSE | 0.3 | 0.31 | 0.013 |
| Far | 0.29 | 0.3 | 0.024 |
| IceW | 0.31 | 0.31 | -0.004 |
| IceN | 0.3 | 0.31 | 0.014 |
| IceE | 0.3 | 0.3 | 0.017 |
| JanM | 0.3 | 0.31 | 0.022 |
| NorS | 0.32 | 0.31 | 0.002 |
| Sval | 0.3 | 0.3 | -0.006 |

**Table S3**. Results from BLASTn runs over the loci sequences against the reference genome of Greenland halibut. For each locus, the chromosome where they were located, as well as the chromosome size, and the start and end positions within the chromosome are indicated. The cover, the e-value and the length of the sequence which was successfully aligned are also indicated. The sex-determining genes, gdf6, sox2 and sox9a2 are included for comparison purposes.

| **Locus** | **Chromosome** | **Chr_size** | **Cover** | **E-val** | **Start** | **End** | **seq_length** |
| --- | --- | --- | --- | --- | --- | --- | --- |
| locus09 | 1 | 31982681 | 1 | 0 | 5879337 | 5882041 | 2704 |
| locus31 | 3 | 28333541 | 0.99 | 0 | 16576346 | 16578856 | 2510 |
| locus30 | 3 | 28333541 | 1 | 0 | 18735005 | 18737995 | 2990 |
| locus58 | 3 | 28333541 | 1 | 0 | 27818486 | 27821157 | 2671 |
| locus22 | 4 | 27977656 | 1 | 0 | 3908957 | 3911956 | 2999 |
| locus23 | 4 | 27977656 | 1 | 0 | 5014577 | 5017574 | 2997 |
| locus50 | 5 | 28081417 | 1 | 0 | 2430278 | 2433271 | 2993 |
| locus64 | 5 | 28081417 | 1 | 0 | 4935930 | 4938925 | 2995 |
| GrHa12 | 5 | 28081417 | 1 | 0 | 5848773 | 5848877 | 104 |
| locus63 | 5 | 28081417 | 1 | 0 | 6446425 | 6449430 | 3005 |
| locus62 | 5 | 28081417 | 1 | 0 | 9312992 | 9315981 | 2989 |
| locus26 | 6 | 26500996 | 1 | 0 | 20424520 | 20427542 | 3022 |
| locus27 | 6 | 26500996 | 1 | 0 | 24345841 | 24347394 | 1553 |
| locus20 | 7 | 27551059 | 1 | 0 | 20923039 | 20926031 | 2992 |
| locus86 | 8 | 26667604 | 1 | 0 | 2519864 | 2522862 | 2998 |
| locus88 | 8 | 26667604 | 1 | 0 | 20664616 | 20667614 | 2998 |
| locus89 | 8 | 26667604 | 0.91 | 0 | 21056990 | 21059723 | 2733 |
| locus90 | 8 | 26667604 | 1 | 0 | 23941012 | 23944002 | 2990 |
| locus15 | 9 | 27987206 | 1 | 0 | 18662725 | 18664911 | 2186 |
| locus14 | 9 | 27987206 | 1 | 0 | 18688236 | 18691239 | 3003 |
| gdf6 | 10 | 24888924 | --- | --- | 6250000 | --- | --- |
| GrHa03 | 10 | 24888924 | 1 | 0 | 6813064 | 6813138 | 74 |
| GrHa14 | 10 | 24888924 | 1 | 0 | 6928053 | 6928157 | 104 |
| locus61 | 10 | 24888924 | 1 | 0 | 7211578 | 7214575 | 2997 |
| GrHa02 | 10 | 24888924 | 1 | 0 | 7257812 | 7257886 | 74 |
| locus60 | 10 | 24888924 | 1 | 0 | 7845793 | 7848789 | 2996 |
| locus59 | 10 | 24888924 | 1 | 0 | 8002904 | 8005897 | 2993 |
| GrHa07 | 10 | 24888924 | 1 | 0 | 9449265 | 9449339 | 74 |
| sox2 | 10 | 24888924 | --- | --- | 10000000 | --- | --- |
| GrHa04 | 10 | 24888924 | 1 | 0 | 12259238 | 12259312 | 74 |
| GrHa18 | 10 | 24888924 | 1 | 0 | 19172840 | 19172914 | 74 |
| GrHa15 | 10 | 24888924 | 1 | 0 | 22204505 | 22204579 | 74 |
| GrHa17 | 10 | 24888924 | 1 | 0 | 23054867 | 23054941 | 74 |
| GrHa06 | 11 | 24491987 | 1 | 0 | 5085921 | 5086025 | 104 |
| locus37 | 12 | 25015410 | 1 | 0 | 17582227 | 17585226 | 2999 |
| locus36 | 12 | 25015410 | 1 | 0 | 18422684 | 18425689 | 3005 |
| locus35 | 12 | 25015410 | 0.99 | 0 | 20028930 | 20030734 | 1804 |
| locus05 | 12 | 25015410 | 0.91 | 0 | 20781708 | 20784429 | 2721 |
| locus56 | 13 | 24611844 | 1 | 0 | 5119336 | 5122320 | 2984 |
| GrHa16 | 13 | 24611844 | 1 | 0 | 14762578 | 14762682 | 104 |
| locus45 | 14 | 27960746 | 1 | 0 | 19454245 | 19457244 | 2999 |

**Table S3 (continued)**. Results from BLASTn runs over the loci sequences against the reference genome of Greenland halibut. For each locus, the chromosome where they were located, as well as the chromosome size, and the start and end positions within the chromosome are indicated. The cover, the e-value and the length of the sequence which was successfully aligned are also indicated. The sex-determining genes, gdf6, sox2 and sox9a2 are included for comparison purposes.

| locus34 | 15 | 24951813 | 1 | 0 | 17775271 | 17778267 | 2996 |
| --- | --- | --- | --- | --- | --- | --- | --- |
| locus33 | 15 | 24951813 | 1 | 0 | 20576225 | 20579207 | 2982 |
| GrHa13 | 16 | 22261345 | 1 | 0 | 13710501 | 13710605 | 104 |
| locus13 | 16 | 22261345 | 1 | 0 | 18119293 | 18122285 | 2992 |
| locus54 | 17 | 21919459 | 1 | 0 | 3519282 | 3522274 | 2992 |
| locus84 | 18 | 21219230 | 1 | 0 | 2102064 | 2105041 | 2977 |
| locus08 | 18 | 21219230 | 1 | 0 | 5315285 | 5318269 | 2984 |
| locus07 | 18 | 21219230 | 1 | 0 | 12102636 | 12105630 | 2994 |
| locus06 | 18 | 21219230 | 1 | 0 | 13299846 | 13302842 | 2996 |
| locus48 | 19 | 19781999 | 1 | 0 | 3552472 | 3555450 | 2978 |
| locus47 | 19 | 19781999 | 1 | 0 | 5042025 | 5045024 | 2999 |
| locus46 | 19 | 19781999 | 1 | 0 | 10930549 | 10933551 | 3002 |
| locus83 | 20 | 21087197 | 1 | 0 | 856738 | 859725 | 2987 |
| locus19 | 20 | 21087197 | 1 | 0 | 14196370 | 14199365 | 2995 |
| GrHa10 | 21 | 20057962 | 1 | 0 | 4032311 | 4032385 | 74 |
| locus75 | 21 | 20057962 | 1 | 0 | 4895085 | 4898036 | 2951 |
| locus74 | 21 | 20057962 | 1 | 0 | 5821463 | 5824444 | 2981 |
| locus73 | 21 | 20057962 | 1 | 0 | 8231219 | 8234197 | 2978 |
| locus68 | 21 | 20057962 | 1 | 0 | 10232518 | 10235517 | 2999 |
| locus67 | 21 | 20057962 | 1 | 0 | 10326535 | 10329529 | 2994 |
| locus66 | 21 | 20057962 | 1 | 0 | 10473989 | 10476996 | 3007 |
| GrHa11 | 21 | 20057962 | 1 | 0 | 10629953 | 10630027 | 74 |
| locus02 | 21 | 20057962 | 1 | 0 | 10773072 | 10776069 | 2997 |
| locus51 | 21 | 20057962 | 1 | 0 | 10841763 | 10844762 | 2999 |
| locus53 | 21 | 20057962 | 1 | 0 | 10925253 | 10928251 | 2998 |
| GrHa01 | 21 | 20057962 | 1 | 0 | 11014839 | 11014913 | 74 |
| locus85 | 21 | 20057962 | 1 | 0 | 11829374 | 11832368 | 2994 |
| locus32 | 21 | 20057962 | 1 | 0 | 12328713 | 12331712 | 2999 |
| locus76 | 21 | 20057962 | 1 | 0 | 12504941 | 12507939 | 2998 |
| GrHa05 | 21 | 20057962 | 1 | 0 | 12831598 | 12831672 | 74 |
| sox9a2 | 21 | 20057962 | --- | --- | 13750000 | --- | --- |
| locus43 | 22 | 18100415 | 1 | 0 | 12929369 | 12932370 | 3001 |
| locus41 | 22 | 18100415 | 1 | 0 | 13647293 | 13650282 | 2989 |
| locus40 | 22 | 18100415 | 1 | 0 | 13647390 | 13650379 | 2989 |
| locus80 | 23 | 15664011 | 1 | 0 | 4145390 | 4148389 | 2999 |
| locus81 | 23 | 15664011 | 1 | 0 | 7163032 | 7166027 | 2995 |
| locus78 | 23 | 15664011 | 1 | 0 | 9678510 | 9681507 | 2997 |
| GrHa08 | 24 | 11085930 | 1 | 0 | 693466 | 693569 | 103 |
| locus17 | 24 | 11085930 | 1 | 0 | 1196042 | 1199023 | 2981 |
| GrHa09 | 24 | 11085930 | 1 | 0 | 4551727 | 4551801 | 74 |
| locus01 | 24 | 11085930 | 1 | 0 | 10576647 | 10579644 | 2997 |
| locus82 | --- | --- | 1 | 0 | 9561 | 12556 | 2995 |

**Table S4**. Pairwise Weir and Cockerham F_ST_ values among sampling locations that were sampled in different years included in this study without separating by sex and including the Pacific sample. Minimum 95% confidence intervals are shown above the diagonal. Bold values indicate statistically significant (2.5% confidence interval > 0) distances.

| **Sample** | IceW18 | IceN18 | IceE18 | IceW19 | IceN19 | IceE19 | JanM18 | JanM19 | JanM20 |
| --- | --- | --- | --- | --- | --- | --- | --- | --- | --- |
| IceW18 | --- | -0.003 | **0.003** | -0.003 | -0.003 | -0.001 | -0.001 | **0.005** | -0.005 |
| IceN18 |  | --- | **0.001** | **0.001** | -0.001 | -0.001 | -0.002 | **0.001** | -0.005 |
| IceE18 | **0.009** | **0.005** | --- | **0.008** | -0.001 | -0.001 | -0.001 | -0.003 | -0.008 |
| IceW19 | 0.002 | **0.006** | **0.018** | --- | **0.003** | **0.005** | **0.004** | **0.01** | **0.002** |
| IceN19 | 0 | 0.001 | 0.005 | **0.009** | --- | -0.004 | -0.004 | **0.001** | -0.006 |
| IceE19 | 0.003 | 0.002 | 0.004 | **0.013** | -0.001 | --- | -0.007 |  | -0.009 |
| JanM18 | 0.004 | 0.002 | 0.007 | **0.012** | -0.001 | -0.003 | --- | -0.004 | -0.009 |
| JanM19 | **0.013** | **0.007** | 0.003 | **0.02** | **0.008** | **0.008** | 0.006 | --- | -0.006 |
| JanM20 | 0 | -0.001 | -0.001 | **0.008** | 0 | -0.003 | -0.002 | 0.001 | --- |

**Table S5**. Pairwise Weir and Cockerham FST values among sampling locations in the North Atlantic for females (above the diagonal) and males (below the diagonal). Bold values indicate statistically significant (α = 0.05) distances. Tables are specified for pairwise comparisons including and excluding SNPs in chromosomes 10 and 21.

| **Sample** | GrlNW | Disko | Davis | GrlSE | Far | IceW | IceN | IceE | JanM | NorS | Sval |
| --- | --- | --- | --- | --- | --- | --- | --- | --- | --- | --- | --- |
| GrlNW | --- | 0.006 | 0 | **0.01** | **0.01** | **0.006** | 0.004 | **0.012** | **0.01** | **0.011** | **0.029** |
| Disko | **0.02** | --- | 0.003 | 0.006 | **0.007** | 0.001 | 0.004 | **0.007** | **0.012** | **0.006** | **0.023** |
| Davis | **0.02** | 0.005 | --- | **0.008** | **0.006** | 0.003 | 0.003 | **0.008** | 0.003 | **0.005** | **0.021** |
| GrlSE | **0.029** | **0.03** | **0.031** | --- | -0.001 | 0.002 | 0.002 | 0.002 | **0.005** | 0.003 | **0.009** |
| Far | **0.07** | **0.113** | **0.103** | **0.053** | --- | 0.001 | 0 | -0.001 | 0.004 | 0.001 | **0.01** |
| IceW | 0.01 | 0.008 | 0.004 | 0.007 | **0.065** | --- | 0.001 | 0.002 | 0.003 | 0.002 | **0.02** |
| IceN | **0.017** | **0.036** | **0.031** | **0.018** | **0.023** | 0.007 | --- | -0.001 | 0.002 | 0.001 | **0.011** |
| IceE | **0.043** | **0.085** | **0.077** | **0.031** | -0.008 | **0.04** | **0.013** | --- | -0.001 | 0 | **0.006** |
| JanM | **0.03** | **0.061** | **0.058** | **0.04** | 0.011 | **0.033** | 0.011 | 0.004 | --- | 0.001 | **0.013** |
| NorS | **0.077** | **0.12** | **0.115** | **0.071** | -0.001 | **0.08** | **0.036** | 0.005 | **0.017** | --- | **0.005** |
| Sval | **0.043** | **0.078** | **0.081** | **0.039** | 0.012 | **0.049** | **0.019** | **0.01** | **0.011** | **0.02** | --- |
| *Excluding sex-associated loci* | | | | | | | | | | | |
| GrlNW | --- | 0.004 | 0.002 | **0.008** | **0.01** | **0.006** | 0.003 | **0.007** | **0.01** | **0.008** | **0.018** |
| Disko | 0.001 | --- | 0.002 | 0.008 | **0.008** | 0.003 | 0.004 | 0.006 | **0.016** | **0.007** | **0.018** |
| Davis | 0.001 | 0.003 | --- | **0.007** | **0.007** | 0.003 | 0.003 | 0.004 | 0.003 | 0.003 | **0.011** |
| GrlSE | 0.009 | 0.01 | **0.016** | --- | -0.003 | 0.002 | 0.001 | 0.002 | **0.006** | 0.003 | **0.007** |
| Far | 0.004 | 0.01 | 0.007 | -0.005 | --- | 0 | 0.001 | -0.002 | 0.004 | 0 | 0.004 |
| IceW | -0.001 | 0.001 | 0.001 | -0.004 | -0.004 | --- | 0.001 | 0 | 0.005 | 0.001 | **0.015** |
| IceN | 0.001 | 0.002 | 0.005 | 0.002 | -0.001 | -0.004 | --- | -0.001 | 0.002 | 0 | 0.006 |
| IceE | 0.012 | **0.017** | 0.016 | -0.008 | -0.007 | -0.001 | 0.009 | --- | -0.003 | 0.001 | 0.005 |
| JanM | 0.004 | 0.001 | 0.005 | 0 | -0.001 | -0.006 | 0 | 0.004 | --- | 0.002 | **0.008** |
| NorS | 0.01 | **0.008** | **0.015** | 0.001 | 0 | 0.002 | 0.005 | 0 | 0.004 | --- | 0.002 |
| Sval | **0.022** | **0.013** | **0.022** | 0.008 | 0.003 | **0.01** | 0.007 | 0.012 | 0 | **0.006** | --- |

**Table S6**. Pairwise Weir and Cockerham F_ST_ values among sampling locations included in this study without separating by sex, excluding sex-associated loci, and including the Pacific sample. Minimum 95% confidence intervals are shown above the diagonal. Bold values indicate statistically significant (2.5% confidence interval > 0) distances.

| **Sample** | Pac | GrlNW | Disko | CanNE | Davis | GrlSE | Far | IceW | IceN | IceE | JanM | NorS | Sval |
| --- | --- | --- | --- | --- | --- | --- | --- | --- | --- | --- | --- | --- | --- |
| Pac | --- | **0.069** | **0.056** | **0.061** | **0.069** | **0.05** | **0.048** | **0.055** | **0.059** | **0.054** | **0.061** | **0.051** | **0.051** |
| GrlNW | **0.105** | --- | -0.001 | **0** | -0.002 | **0.004** | **0.005** | **0.001** | **0.001** | **0.003** | **0.003** | **0.004** | **0.012** |
| Disko | **0.089** | 0.004 | --- | -0.002 | -0.001 | **0.002** | **0.002** | **0** | -0.001 | **0.002** | **0.004** | **0.002** | **0.006** |
| CanNE | **0.108** | **0.013** | 0.006 | --- | -0.001 | **0.004** | **0.002** | -0.005 | -0.001 | **0.005** | **0.006** | **0.001** | **0.007** |
| Davis | **0.104** | 0 | 0.003 | 0.01 | --- | **0.004** | **0.005** | **0** | **0** | **0.004** | **0.002** | **0.003** | **0.01** |
| GrlSE | **0.08** | **0.009** | **0.008** | **0.016** | **0.01** | --- | -0.005 | -0.002 | -0.002 | -0.002 | -0.001 | -0.001 | **0.003** |
| Far | **0.079** | **0.011** | **0.008** | **0.015** | **0.01** | -0.003 | --- | -0.002 | -0.002 | -0.003 | -0.001 | -0.002 | **0.002** |
| IceW | **0.086** | **0.004** | **0.003** | 0.005 | **0.003** | 0.001 | 0 | --- | -0.002 | -0.002 | **0** | -0.001 | **0.007** |
| IceN | **0.09** | **0.004** | 0.003 | 0.009 | **0.003** | 0 | 0 | -0.001 | --- | -0.002 | -0.001 | -0.001 | **0.004** |
| IceE | **0.089** | **0.009** | **0.009** | **0.017** | **0.008** | 0.001 | -0.001 | 0.001 | 0 | --- | -0.002 | -0.001 | **0.003** |
| JanM | **0.094** | **0.009** | **0.011** | **0.015** | **0.007** | 0.003 | 0.002 | **0.003** | 0.002 | 0 | --- | **0** | **0.003** |
| NorS | **0.082** | **0.009** | **0.008** | **0.011** | **0.007** | 0.001 | 0 | 0.002 | 0.001 | 0.001 | **0.002** | --- | **0.002** |
| Sval | **0.093** | **0.021** | **0.016** | **0.019** | **0.016** | **0.007** | **0.005** | **0.012** | **0.007** | **0.008** | **0.007** | **0.004** | --- |


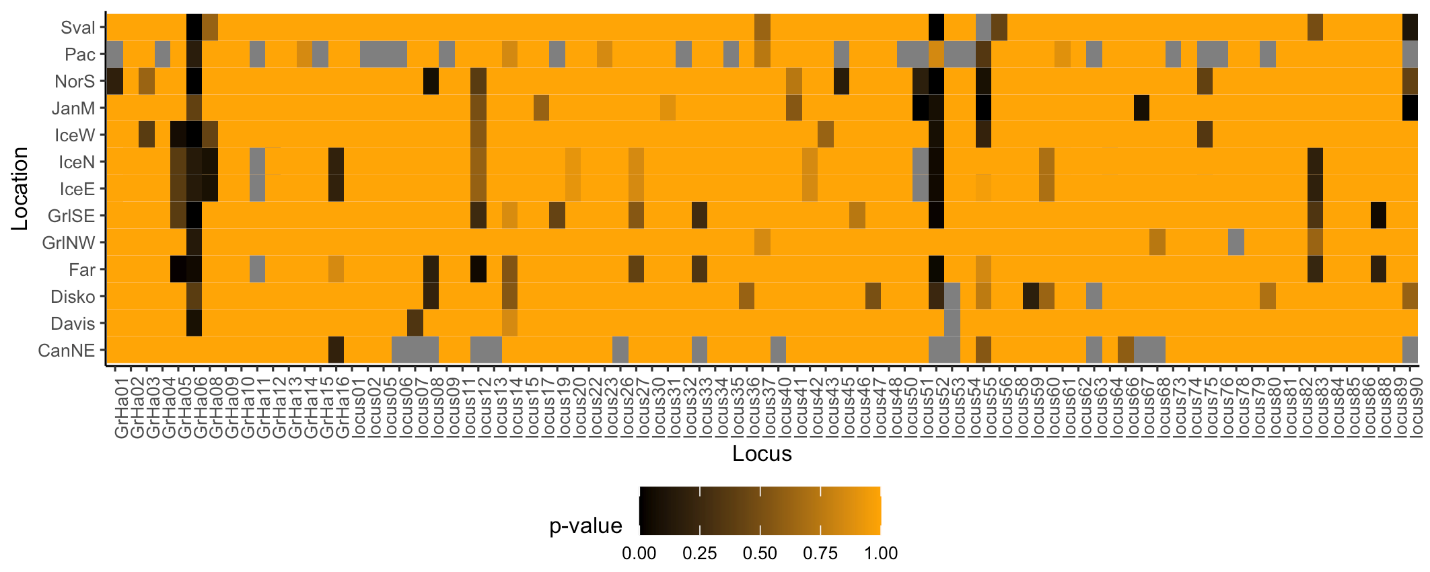


**Figure S1**. Results for Hardy-Weinberg Equilibirum (HWE) tests for deficit of heterozygotes across locations sampled and loci genotyped. The P value is shown in a color scale with orange equivalent to p = 1 and black p < 0.00001; grey color is equivalent to monomorphism. The P values were estimated with a Markov chain algortihm (see main text).


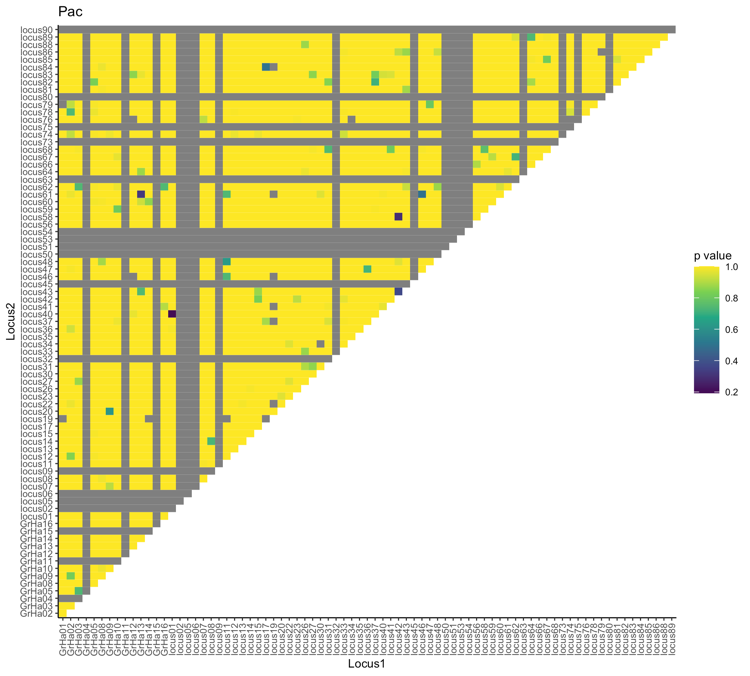

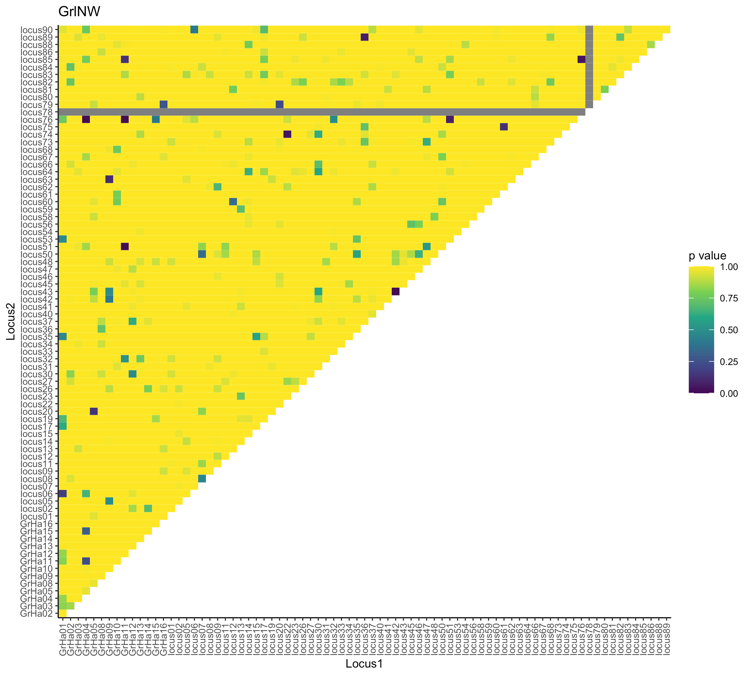

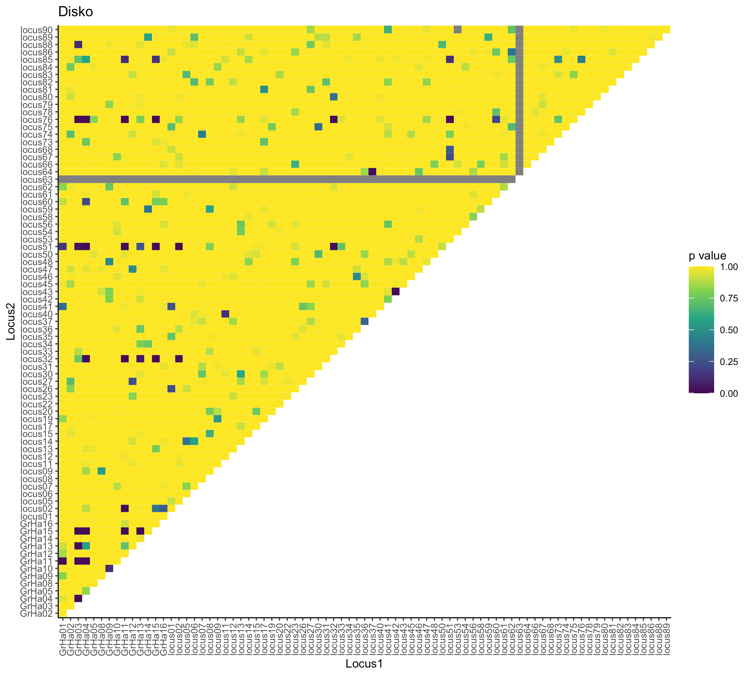

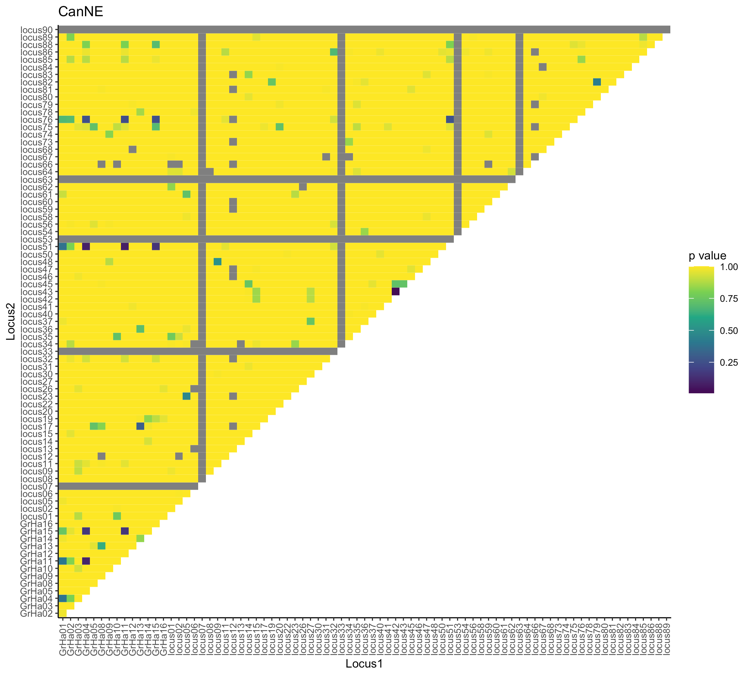
**Figure S2**. Linkage disequilibrium tests for all loci genotyped across all locations sampled for Greenland halibut that passed missing data, minor allele frequency and Hardy-Weinberg Equilibrium tests. P values were corrected by false discovery rate method with darker colors indicating lower p values.


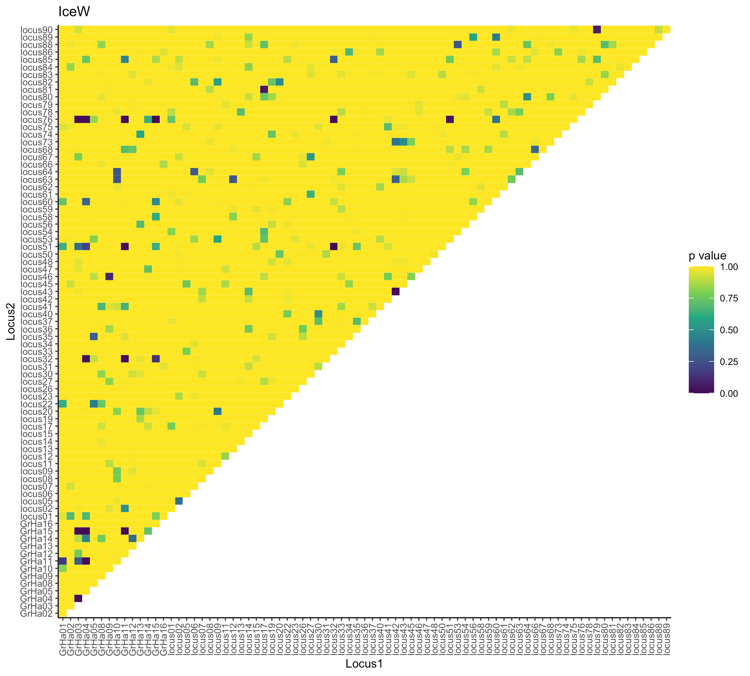

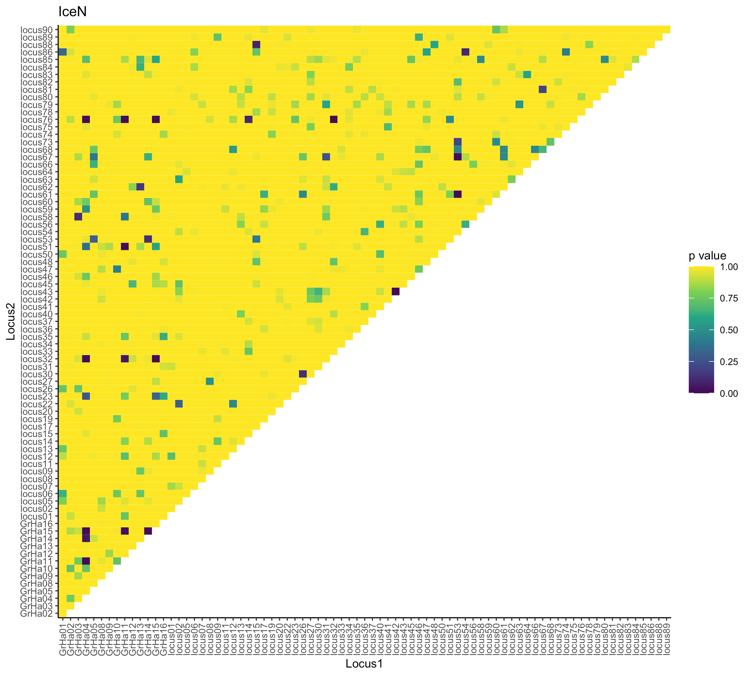

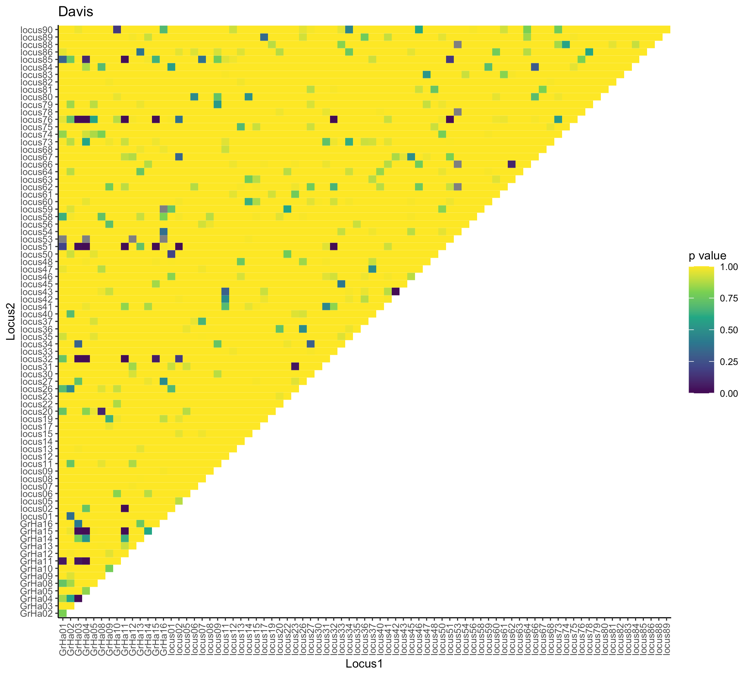

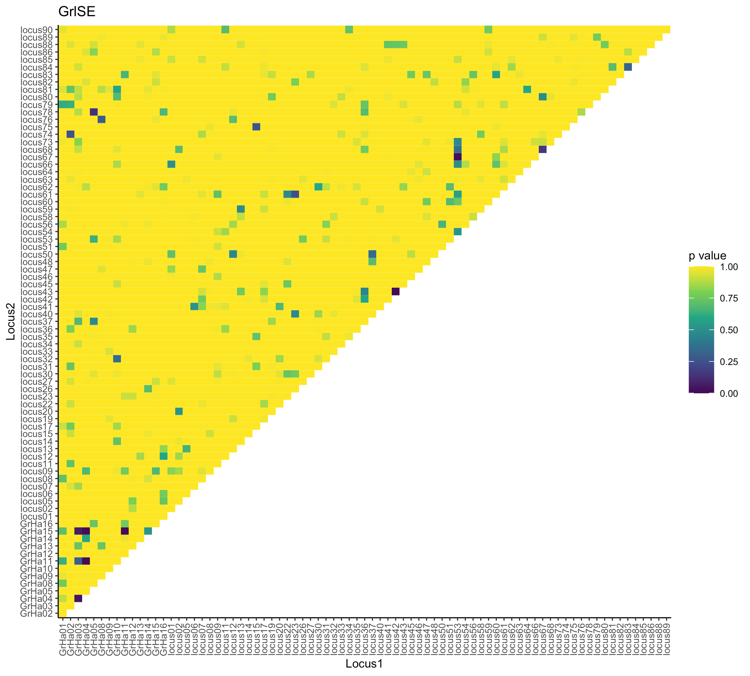
**Figure S2 (continued)**. Linkage disequilibrium tests for all loci genotyped across all locations sampled for Greenland halibut that passed missing data, minor allele frequency and Hardy-Weinberg Equilibrium tests. P values were corrected by false discovery rate method with darker colors indicating lower p values.


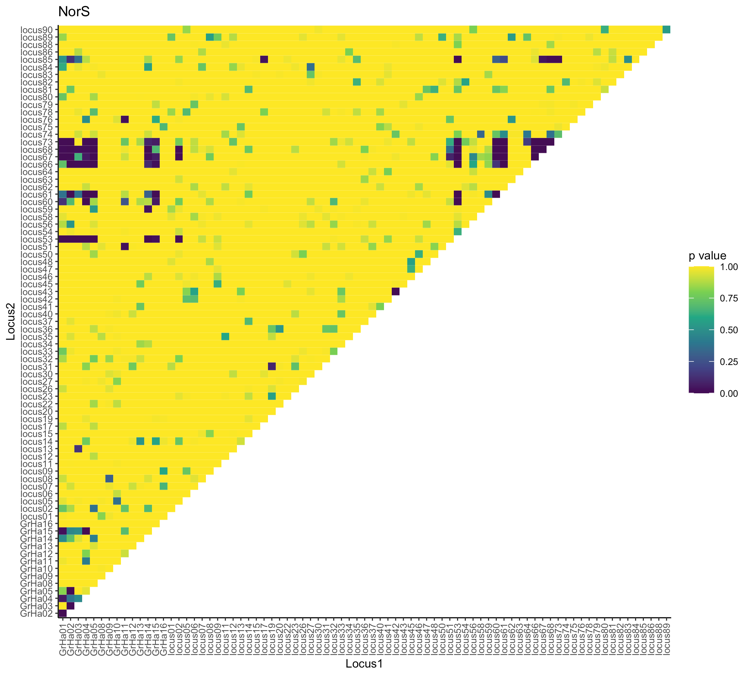

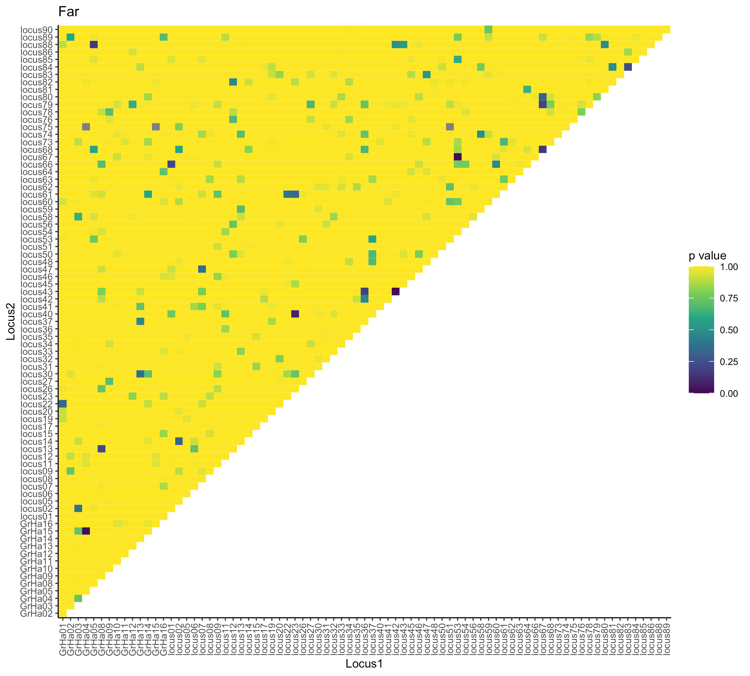

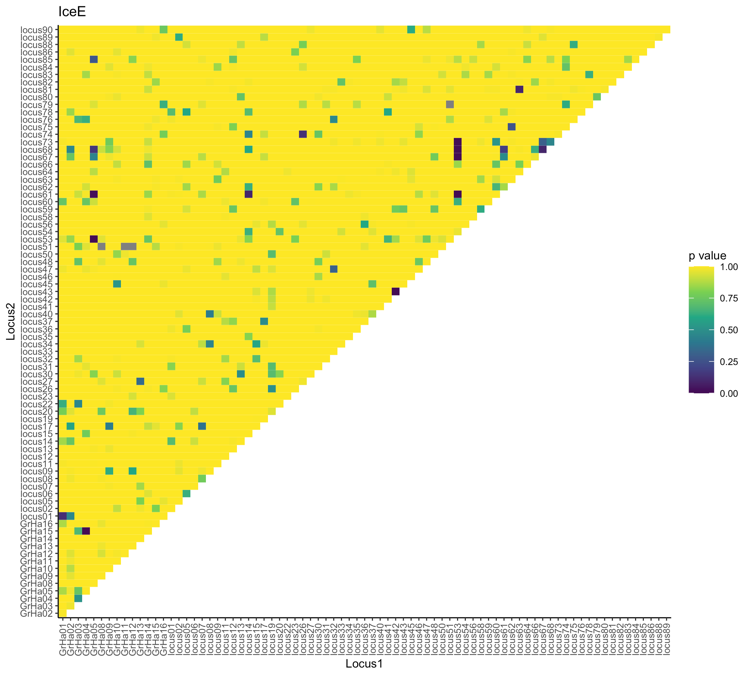

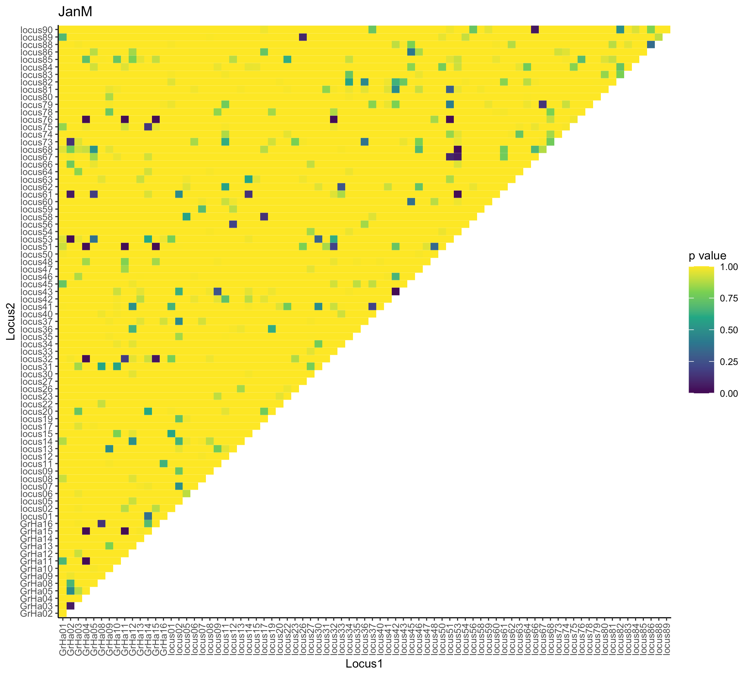


**Figure S2 (continued)**. Linkage disequilibrium tests for all loci genotyped across all locations sampled for Greenland halibut that passed missing data, minor allele frequency and Hardy-Weinberg Equilibrium tests. P values were corrected by false discovery rate method with darker colors indicating lower p values.


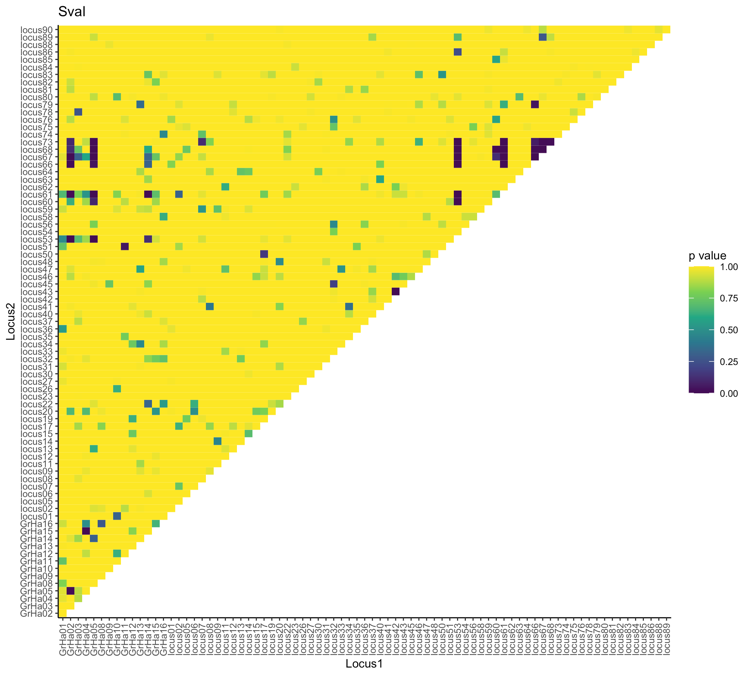


**Figure S2 (continued)**. Linkage disequilibrium tests for all loci genotyped across all locations sampled for Greenland halibut that passed missing data, minor allele frequency and Hardy-Weinberg Equilibrium tests. P values were corrected by false discovery rate method with darker colors indicating lower p values.


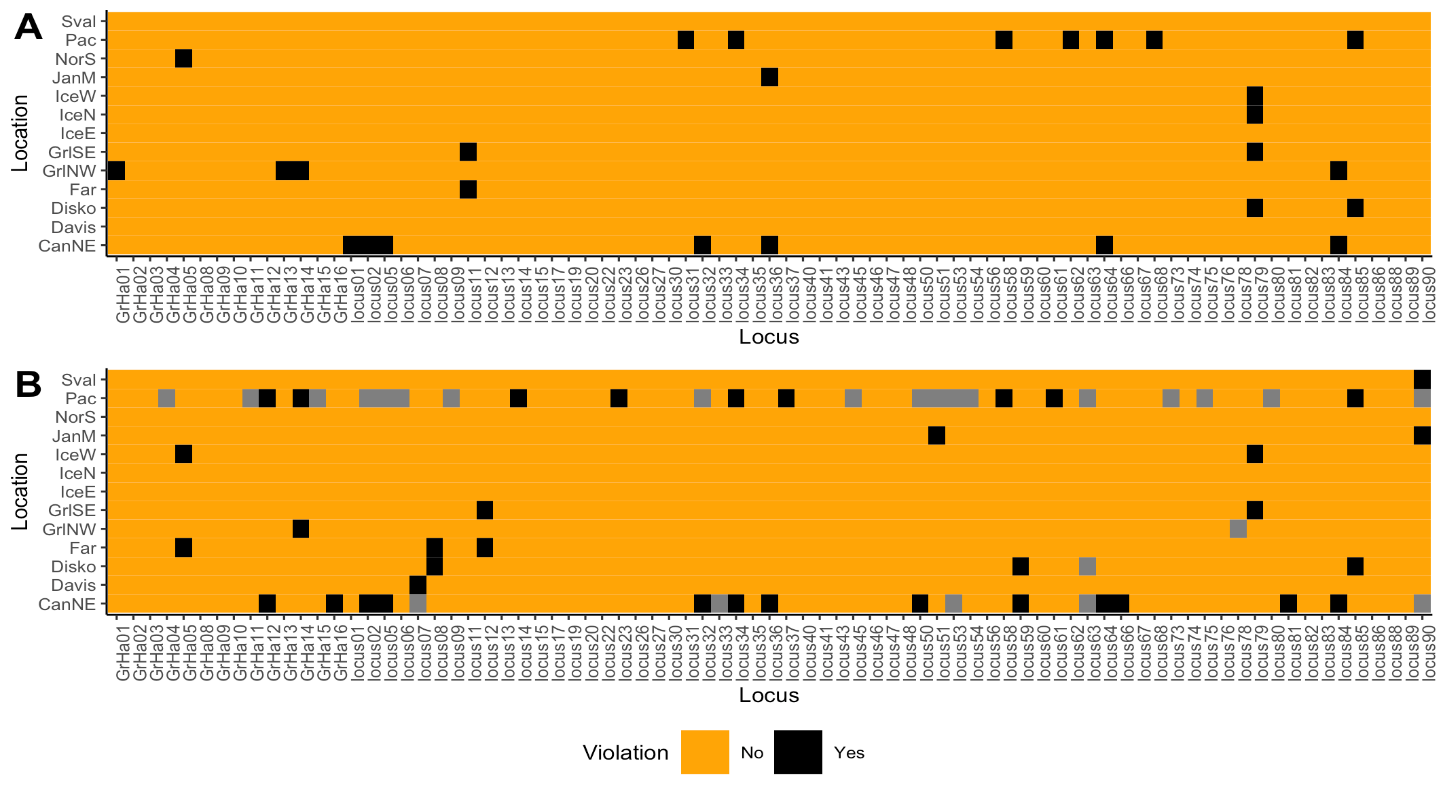


**Figure S3**. Overview of observed heterozygosity (A) and F_IS_ (B) violations (see main text) across locations sampled and loci genotyped for Greenland halibut. Values not conforming with established thresholds (A: H_O_ < 0.5; B: -0.40 < F_IS_ < 0.40) are indicated in black, the rest in orange. Estimates in grey were not possible to calculate.


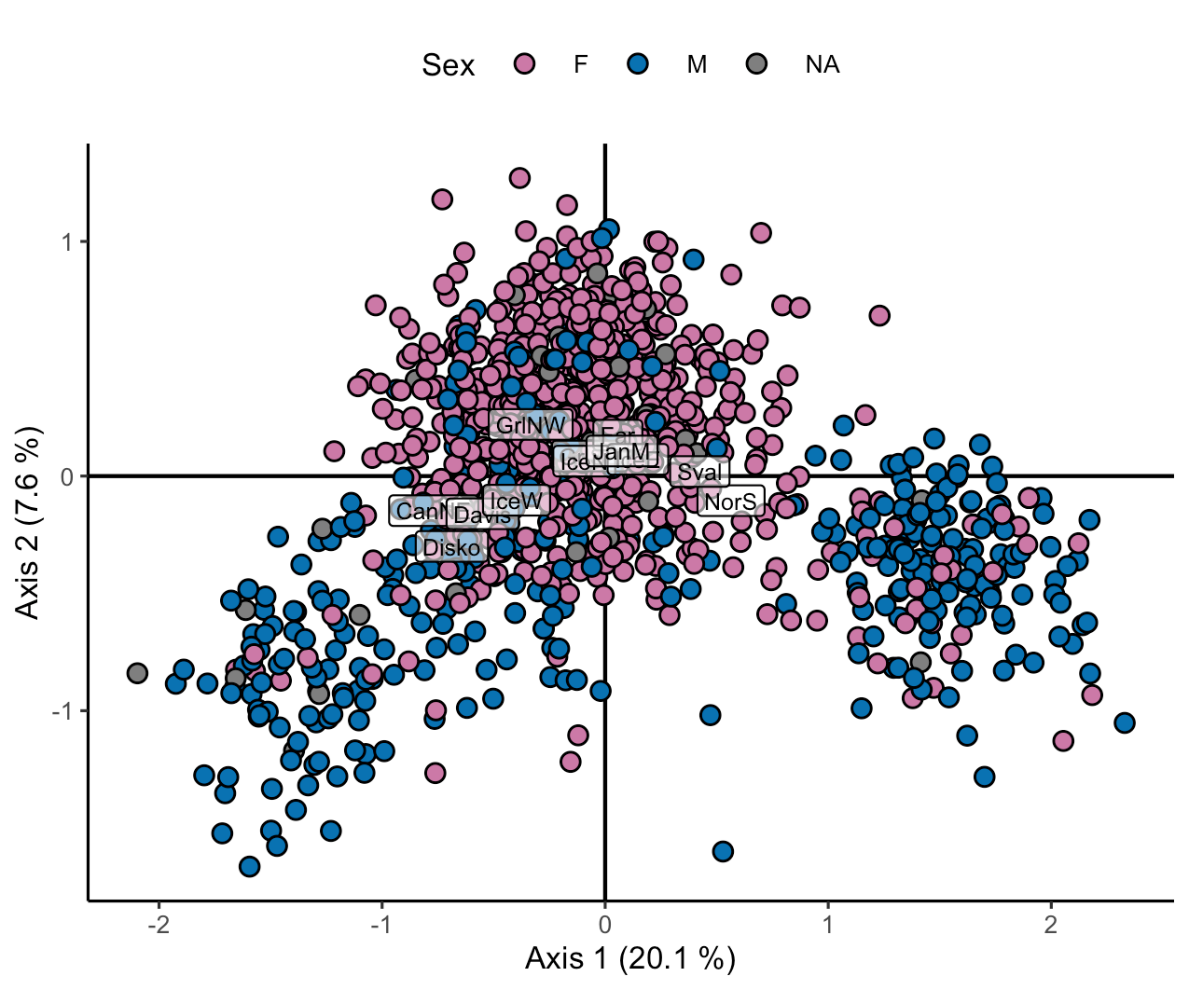


**Figure S4**. Principal coordinate analysis plots based on Euclidian distances using the datasets with only the sex-associated loci. Location labels are the coordinates mean calculated from individuals within locations. Dots are colored by sex, notice that individuals that were not visually sexed are also included.


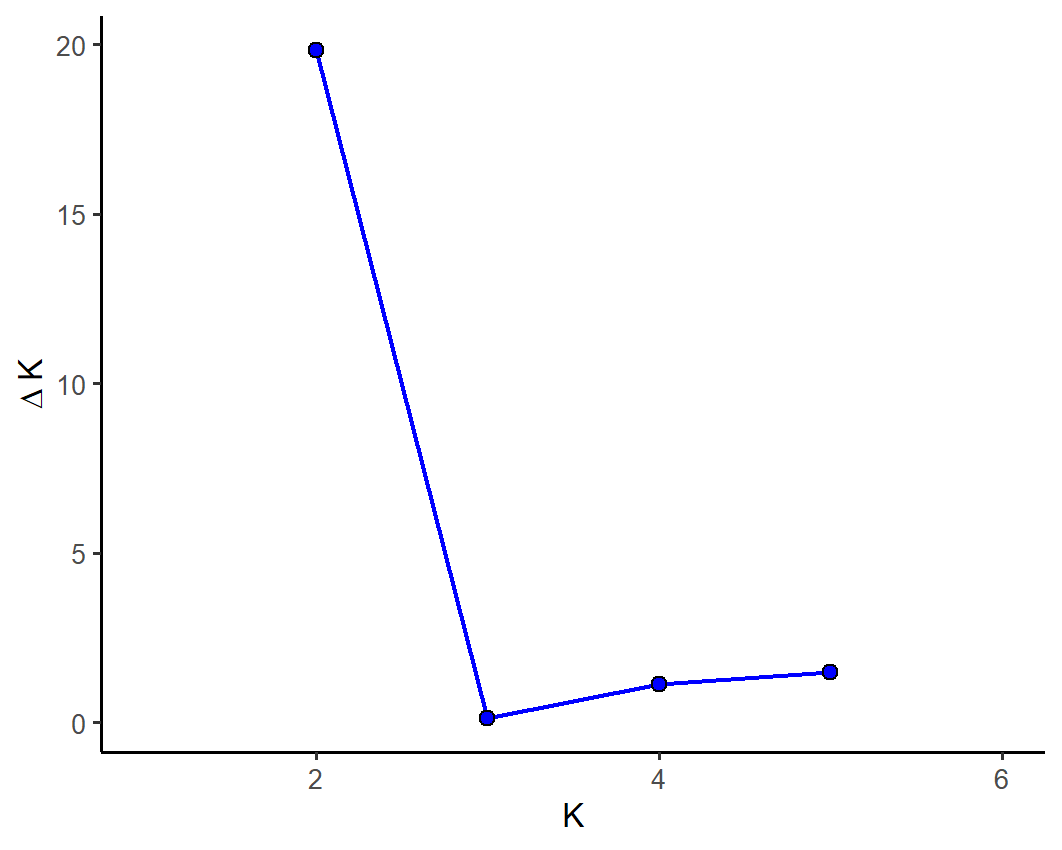


**Figure S5**. Delta K plot of the Evanno test.


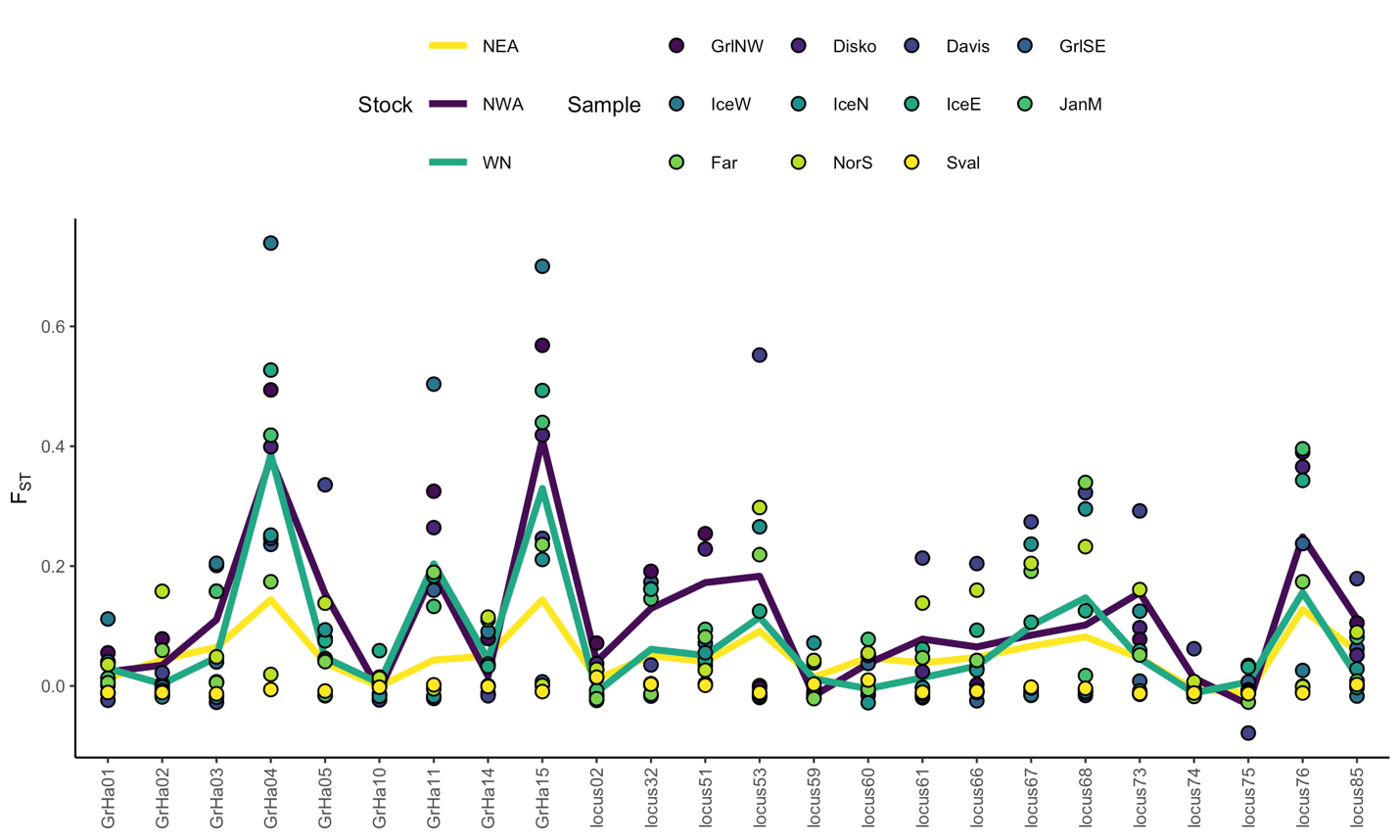


**Figure S6**. F_ST_ values per each of the sex-associated locus (those located in chromosomes 10 and 21) within each location. The averages are shown for a classification of the locations into three main offshore stocks: NWA (GrlNW, Disko and Davis), WN (GrlSE, IceW, IceN, IceE and Far) and NEA (JanM, NorS and Sval). See the main text or Table 1 for the acronyms of the different locations.
